# Tailored Black Phosphorus for Erythrocyte Membrane Nanocloaking with Interleukin-1*α* siRNA and Paclitaxel for Targeted, Durable, and Mild Combination Cancer Therapy

**DOI:** 10.7150/thno.37123

**Published:** 2019-09-19

**Authors:** Wenquan Ou, Jeong Hoon Byeon, Zar Chi Soe, Bo Kyun Kim, Raj Kumar Thapa, Biki Gupta, Bijay Kumar Poudel, Sae Kwang Ku, Chul Soon Yong, Jong Oh Kim

**Affiliations:** 1 College of Pharmacy, Yeungnam University, Gyeongsan 38541, Republic of Korea.; 2 School of Mechanical Engineering, Yeungnam University, Gyeongsan 38541, Republic of Korea.; 3 College of Korean Medicine, Daegu Haany University, Gyeongsan 38610, Republic of Korea.

**Keywords:** therapeutic nanosystems, tailored black phosphorus nanoparticles, erythrocyte membranes, core@shell constructs, combination cancer therapies.

## Abstract

Several therapeutic nanosystems have been engineered to remedy the shortcomings of cancer monotherapies, including immunotherapy (stimulating the host immune system to eradicate cancer), to improve therapeutic efficacy with minimizing off-target effects and tumor-induced immunosuppression. Light-activated components in nanosystems confer additional phototherapeutic effects as combinatorial modalities; however, systemic and thermal toxicities with unfavorable accumulation and excretion of nanoystem components now hamper their practical applications. Thus, there remains a need for optimal multifunctional nanosystems to enhance targeted, durable, and mild combination therapies for efficient cancer treatment without notable side effects.

**Methods:** A nanosystem constructed with a base core (poly-L-histidine [H]-grafted black phosphorus [BP]) and a shell (erythrocyte membrane [EM]) is developed to offer a mild photoresponsive (near-infrared) activity with erythrocyte mimicry. In-flight electrostatic tailoring to extract uniform BP nanoparticles maintains a hydrodynamic size of <200 nm (enabling enhanced permeability and retention) after EM cloaking and enhances their biocompatibility.

**Results:** Ephrin-A2 receptor-specific peptide (YSA, targeting cancer cells), interleukin-1α silencing small interfering RNA (ILsi, restricting regulatory T cell trafficking), and paclitaxel (X, inducing durable chemotherapeutics) are incorporated within the base core@shell constructs to create BP-H-ILsi-X@EM-YSA architectures, which provide a more intelligent nanosystem for combination cancer therapies.

**Conclusion:** The in-flight tailoring of BP particles provides a promising base core for fabricating <200 nm EM-mimicking multifunctional nanosystems, which could be beneficial for constructing smarter nanoarchitectures to use in combination cancer therapies.

## Introduction

There are several limitations in current cancer therapies, such as excessive cytotoxicity, metastasis, tumor recurrence, and resistance to conventional cancer chemotherapies, which created the need to create alternative or combination therapeutic modalities for safer and more efficacious cancer treatment 1,2. Nanosystems consisting of polymers, micelles, or liposomes have been intensively adopted to offer tumor-selective delivery of chemotherapeutic agents with enhanced permeability and retention (EPR) for preferential accumulation and efficacy 3. Cancer immunotherapy, which activates the body's immune system to enhance therapeutic efficacy while minimizing the adverse effects of treatment, has also been recently investigated to overcome the adverse effects of traditional cancer monotherapies 4. Immune checkpoint inhibitors, adjuvants, or vaccines have improved antitumor immune responses in many preclinical settings 5; however, nanosystems are required to deliver tumor-selective immunostimulatory agents together with cancer chemotherapies 6, which signifies that engineering for suitable nanosystems might play an important role in cancer therapy because of their ability to load chemotherapeutic/immunostimulatory agents, target tumors, provide an EPR effect, and assign a stimuli-responsive activity to maximize therapeutic efficacy 7-9.

Combining phototherapy (using near-infrared [NIR]-activated nanosystems) with chemotherapy (as chemophototherapy) or immunotherapy (as photoimmunotherapy) has been frequently used to release anticancer drugs or endogenous immunostimulatory molecules in response to NIR irradiation in order to improve anticancer drug bioavailability or strengthen anticancer immune responses 3,6. Carbon (graphene and carbon nanotubes)- and metal (gold nanostructures and metal sulfides)-based nanosystems have been exploited as phototransducers for NIR-induced combination cancer therapies 10,11. Despite their promising noninvasive phototherapeutic effects, developing biodegradable and long-term systemically nontoxic nanosystems *in vivo* is a big hurdle for further clinical application because of the carbon-induced oxidative stress and inflammation and metal-related cytotoxicities 11-13. More recently, even though biodegradable (*e.g.*, black phosphorus [BP] and copper sulfide) and disintegratable (*i.e.*, nanoclusters of ultrasmall inorganic particles) phototransducing nanosystems have been introduced to resolve these issues 14,15, precise and complex designs are needed to modulate biodegradation kinetics and confer assembly-disassembly functions for effective, durable, and tumor-specific therapies that are biosafe 16,17.

Thermal harshness from photoirradiation onto nanosystems 18-20 and undesirable rapid clearance of nanosystems by the immune system 21-23 are additional issues in the clinical translation of light-activated combination cancer therapy. Even though the high temperatures (Δ*T* [temperature elevation] >20^o^C, higher than the basal body temperature) of the nanosystems produced by photoirradiation can induce significant anticancer effects, these temperatures can cause the necrosis of surrounding healthy cells, as well as burning, blistering, and pain. On the other hand, moderate temperatures (Δ*T* ≤ 7^o^C; *i.e.*, fever-like) from mild photoirradiation have been reported to be appropriate for modulating drug-resistance-related genes and promoting thermal tolerance of extracellular heat shock proteins 24,25. In addition, these temperatures achieved an immunofavorable tumor microenvironment that is suitable for enhancing the immunotherapeutic efficacy 25,26. Biomimetic functionalization of nanosystems to secure their effective accumulation at the tumor site by bypassing macrophage uptake and prolonging blood circulation has also been attempted to further enhance combination cancer therapy 27-29. Cell membrane-camouflaged nanosystems have also been used for imaging specific tumor and preventing viral infection 30,31. Erythrocytes, lymphocytes, macrophages, or their artificial constructs have been used to ensure the biomimicry of developed nanosystems because of their intrinsic biocompatibility, biodegradability, and nonimmunogenicity 32,33. In particular, the use of erythrocytes membranes (EMs) with nanosystems has become a representative platform for naturally enhancing the ability to increase immune responses, load chemotherapeutic and/or immunostimulatory agents, target and penetrate tumors, and modify surfaces, resulting in reduced doses of therapeutic agents and systemic side effects 34,35. Nevertheless, developing optimal processes (chemistry, manufacturing, and control) and architectures (size, morphology, surface, and flexibility) to generate the desired nanosystems might also be a challenging cue for researchers to conduct clinical trials on combination cancer therapies 6,36-38.

**Figure 1:**
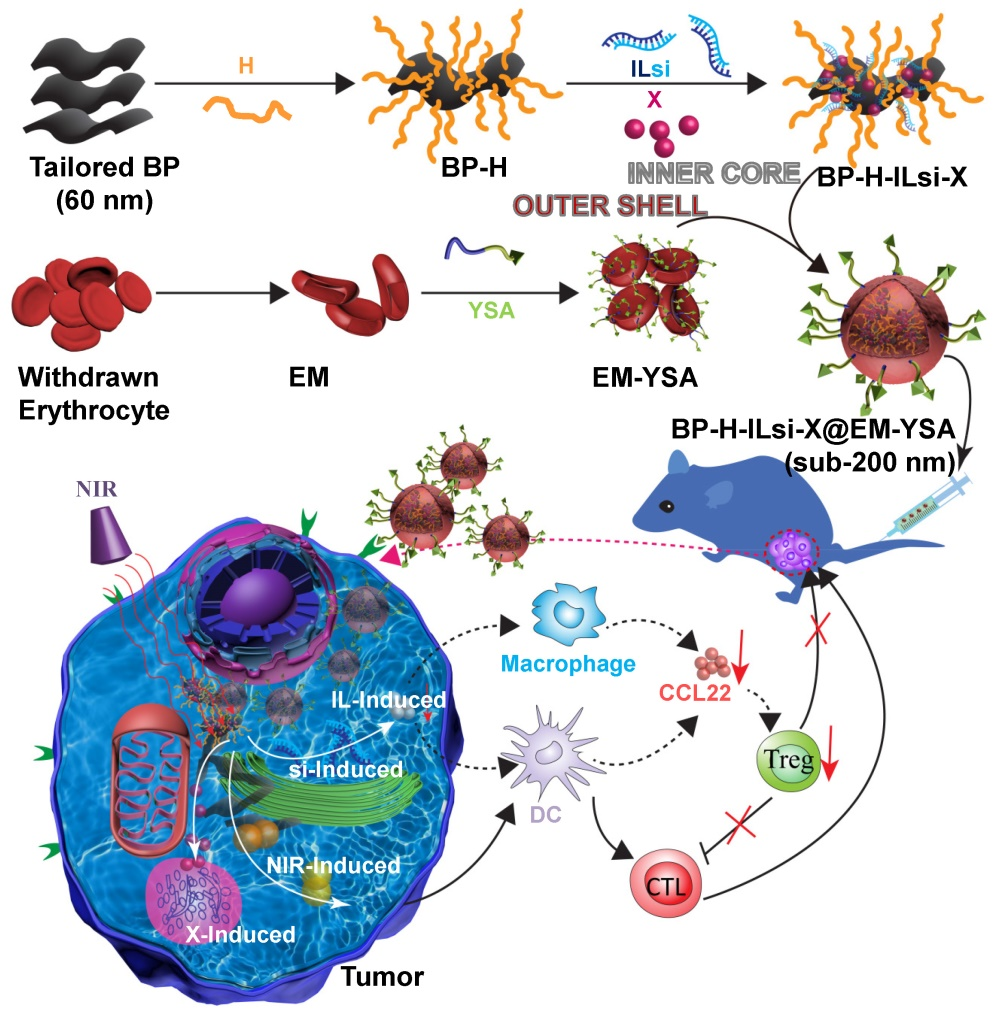
** A schematic of the preparation of the BP-H-ILsi-X@EM-YSA core@shell architecture and concept of combination immunotherapy against tumors.** BP nanoparticles tailored from electrostatic size classification were used directly for grafting with H and incorporated further with ILsi and X to prepare the inner core constructs (BP-H-ILsi-X). EMs isolated from the blood of C57BL/6 mice were incubated with pristine YSA and DSPE-PEG_2000_-COOH and product (YSA-PEG_2000_-DSPE) for anchoring YSA onto the EM to prepare the outer shell constructs (EM-YSA). These two components were coextruded through a membrane filter (200 nm cutoff) as BP-H-ILsi-X@EM-YSA core@shell nanosystems (<200 nm) for intravenous injection. Finally, the antitumor activity of the nanosystems combined with NIR irradiation to enhance the chemophotoimmunotherapeutic effect was evaluated.

To this end, we designed and prepared ephrin-A2 receptor-specific peptide (YSA) anchored EM-cloaked all-in-one nanosystems (<200 nm, EPR-enabled) in order to ensure targeted, durable, and mild cancer chemophotoimmunotherapy without notable side effects. The nanosystems were constructed using a “core@shell” architecture, where poly-L-histidine (H, cross-linking agent) grafted tailored (as 60 nm) BP (NIR-stimulus core) was incorporated with interleukin-1*α* silencing small interfering RNA (ILsi) and paclitaxel (X) as the inner core, whereas YSA-anchored EM was used as the outer shell, resulting in the creation of BP-H-ILsi-X@EM-YSA nanosystems (**Figure 1**). In order to achieve a <200 nm architecture, coarse BP flakes were in-flight-tailored through a series connection of probe sonication, mechanical spraying, and electrostatic size classification (**Figure S1**A). Specifically, singly charged positive BP particles (geometric mean diameter [GMD] = 60.0 nm, geometric standard deviation [GSD] = 1.08) were extracted from mechanically spray-dried BP particles (GMD = 61.3 nm, GSD = 1.55) using a nanodifferential mobility analyzer (NDMA) (**Figure S1**B), and the classified BP particles were electrostatically collected in powder form on a cylindrical aluminum rod with a negative electrical potential. These tailored BP particles were more morphologically uniform (**Figure S1**C) and biocompatible (**Figure S1**D) than untailored BP particles and were directly applied for grafting H and subsequently loading ILsi and X to form BP-H-ILsi-X without further purification. BP-H-ILsi-X was then cloaked with EM-YSA by extruding the mixture of BP-H-ILsi-X and EM-YSA through a miniextruder with a 200 nm polycarbonate membrane to form <200 nm BP-H-ILsi-X@EM-YSA core@shell nanosystems (*i.e.*, much smaller compared to native erythrocytes) for enhancing their extravascular diffusion into tumor mass after intravenous injection 39. The EM-YSA configuration was selected in this study not only because YSA anchoring of the nanosystems enhanced their tumor-specific trafficking and penetration 40, but also because EM cloaking suppressed rapid BP degradation/clearance during prolonged circulation 41,42. The EM-YSA configuration further improved the biocompatibility of the BP particles (**Figure S1**D) and promoted a mild hyperthermic microenvironment (Δ*T* ≤ 9^o^C at 808 nm, 0.5 W/cm^2^, 5 min, **Figure S1**E) for the structural and functional stability of the therapeutic agents. Successive loading of ILsi and X on the tailored BP core for EM cloaking was motivated by the potent gene-silencing effect of si 43 with the inhibition of IL (a promoter of inflammation) secretion 44 and the steady-release characteristics of hydrophobic X 45 for collectively providing efficient cancer chemophotoimmunotherapy.

## Methods

### Tailoring Coarse BP Flakes

As illustrated in **Figure S1**A, coarse BP flakes (7723-14-0; ACS Material, USA) were first dispersed in deoxygenated water, and the dispersion was then poured into a reactor on a plate-type Peltier cooler (at -20^o^C, preventing reactor overheating) equipped with a VCX 750 probe sonicator (Sonics & Materials, USA). The sonicated dispersion was then connected to a peristaltic pump (Watson-Marlow Fluid Technology Group, USA) to continuously supply the dispersion into an AG-01 collison-type atomizer (HCT-m, Republic of Korea) for mechanical spraying. The sprayed dispersion (*i.e.*, droplets containing the pulverized BP particles) in nitrogen gas flow subsequently entered a diffusion dryer (eliminating residual water) and an XRC-05 soft X-ray charger (HCT-m, Republic of Korea; creating Boltzmann charge distribution). The BP particle-laden flow was then entered into a NDMA (3085, TSI, USA) equipped with a 3082 electrostatic classifier (TSI, USA) to control the NDMA operation for in-flight extraction of the selected size of 60 nm (the equivalent mobility diameter of positively singly charged particles) for the BP particles from the inflowing BP particles. The tailored BP particles were finally collected on a polished aluminum rod with a negative electrical potential (2.5 kV/cm).

### Assembly of BP-H-ILsi-X @EM-YSA Core@Shell Nanosystems

In order to construct the inner core of the nanosystem, 5 mg of tailored BP particles was dispersed into 10 mL of deoxygenated water under bath sonication for 30 min. H (*Mw* = 5000-25000; Sigma-Aldrich, USA; 20 *w*/*w* [H/BP]%) was added to the dispersion and stirred for 3 h for grafting onto the BP surface as BP-H. A 50 nM murine ILsi (Santa Cruz Biotechnology, USA) was then loaded onto the BP-H using electrostatic attraction for overnight incubation, and X (15 *w*/*w*% in the nanosystem; LC Laboratories, USA) was further added to the dispersion and incubated for another 6 h to form BP-H-ILsi-X. Finally, this reacted dispersion was purified using an Amicon^®^ 100 kDa MWCO centrifugal filter unit (Merck Millipore, USA). For the outer shell of the nanosystem, blood was first drawn from healthy C57BL/6 mice and centrifuged (1000×*g*) at 4^o^C for 5 min to remove the serum before adding a hypotonic medium (0.25× PBS) for hemolysis (performed on ice for 1 h). The solution was centrifuged again at 1000×*g* at 4^o^C for 5 min to remove the hemoglobin. The resulting pinkish pellet (EM) was washed with and stored in PBS at 4^o^C for anchoring YSA. In order to confer YSA's anchoring function (YSAYPDSVPMMS, *Mw* = 1347.52; Bioneer, Republic of Korea), 0.05 mM YSA was added to a 0.02 mM NHS-PEG_2000_-DSPE (Nanosoft Polymers, USA) solution containing 0.1 M MES buffer (pH 7.0, RES0113M-A7; Sigma-Aldrich, USA) under magnetic stirring at room temperature for 4 h to form YSA-PEG_2000_-DSPE (anchorable YSA). The excessive reactants were removed using a 3500 kDa MWCO dialysis bag (Spectrum Chemical, USA) in distilled water for 72 h. The freeze-dried YSA-PEG_2000_-DSPE was then added to EM solution and incubated for 30 min to form EM-YSA as the outer shell. EM-Scr was prepared by replacing YSA with Scr (PPMMASSYYDVS, *Mw* = 1347.52; Bioneer, Republic of Korea) to form Scr-PEG_2000_-DSPE for comparisons. The inner core and outer shell constructs were finally coextruded through a 200 nm polycarbonate membrane-installed miniextruder (Avanti Polar Lipids, USA) 21 times to fabricate the BP-H-ILsi-X@EM-YSA core@shell nanosystems.

### Physicochemical Characterizations

In-flight tailoring of BP flakes was examined using the 3936 scanning mobility particle sizer (TSI, USA) by comparing the size distributions between the nonclassified and size-classified cases. The DLS size distributions and zeta potentials of the dispersed particles were measured using the Nano-S90 Zetasizer (Malvern Instruments, UK). The morphologies of the nanosystems (stained with 1% uranyl acetate for the instrumentation), including individual BP and EMs were observed using Tecnai G^2^ F20 S-TWIN TEM (FEI, USA). In order to evaluate the optimal N/P ratio, an agarose gel electrophoresis assay using 1.5 % agarose gel (Invitrogen, USA) containing GelRed™ nucleic acid stain (Biotium, USA) was used with modulating BP-H and ILsi contents. SDS-PAGE electrophoresis using 10% polyacrylamide gel with Coomassie brilliant blue stain (Imperial™ Protein Stain, Thermo Fisher Scientific, USA) was used to confirm EM cloaking by observing the existence of the membrane protein on the inner core. EDS using INCA 350 (Oxford Instruments, UK) was further conducted to verify cloaking by comparing elemental compositions. The surface chemistries and crystalline properties of the nanosystem were examined using the Nicolet Nexus 670 FTIR system (Thermo Fisher Scientific, USA) and D/MAX-2500 XRD system (Rigaku, Japan) measurements, respectively.

The generation of O=O was estimated by grading DPBF oxidation (monitoring absorbance at 410 nm) after treatment with the nanosystem, including naked BP (100 µg/mL basis of BP concentration) under NIR irradiation (808 nm, 0.5 W/cm^2^) for 5 min. The *EE* and *LC* of X were determined using a CM5000 high-performance liquid chromatography (HPLC) system (Hitachi, Japan) equipped with a C18 column (250 × 4.6 mm, 5 μm; GL Sciences, USA). The absorbance of X at 227 nm was monitored using a U-2800 UV-Vis spectrophotometer (PerkinElmer, USA) under an eluting condition (in a ratio of distilled water 4 and acetonitrile 6) with a flow rate of 1 mL/min (dynamic condition). *In vitro* X release from the nanosystem, including BP-H-ILsi-X (for comparison), was examined using the dynamic dialysis method. Specifically, 1 mL nanosystem or BP-H-ILsi-X containing 15 *w*/*w*% X was added to a dialysis bag and placed into a shaking incubator (37^o^C, 100 rpm) filled with 30 mL of PBS buffer (pH 6.5). The released X content was determined using HPLC. The stabilities in size and X content of the nanosystem during shaking incubation (37^o^C, 100 rpm) in different media (distilled water, PBS, or mouse serum) were determined using Zetasizer and HPLC systems, respectively. In addition, ILsi stability in the serum was verified using 1.5% agarose gel electrophoresis and subsequent incubation for 10 h.

### *In vitro* Cellular Uptake and Endosomal Escape of ILsi

In order to examine the intracellular uptake of the nanosystem, MC-38 cells (Kerafast, USA) were cultured on a glass slide placed in a 12-well plate at a density of 5 × 10^4^ cells/well. After incubating for 24 h, the cells were treated with the nanosystem that included 50 nM Cy5-labeled ILsi (Cy5-ILsi; GenePharma, China) (BP-H-Cy5-ILsi-X@EM-YSA), including the ILsi-labeled BP-H-X@EM-Scr and Lipo (Lipofectamine® 2000; Thermo Fisher Scientific, USA) for 6 h for comparison. The supernatant was discarded, substituted with PBS, and fixed with 4% paraformaldehyde. After 10 min, the cells were further washed with PBS for CLSM (Nanoscope, Republic of Korea) observation to track the internalization of the nanosystem. Uptake quantification was determined using the FACSCalibur™ flow cytometry system (BD Biosciences, USA).

In order to determine the endosomal escape of ILsi, MC-38 cells (5 × 10^4^ cells/well) were cultured on a glass slide and placed in a 12-well plate. After incubating for 24 h, the cells were treated with the nanosystem that included 50 nM FAM-labeled ILsi (FAM-ILsi; GenePharma, China) (BP-H-FAM-ILsi-X@EM-YSA) for 4 h in the absence and presence of subsequent NIR irradiation (808 nm, 0.5 W/cm^2^, 5 min). After incubating for an additional 2 h, the cells were washed with PBS and fixed with 4% paraformaldehyde for 10 min. The cells were then stained with DAPI and LysoTracker Red to comparatively observe the localization of ILsi using CLSM.

### *In vitro* Gene Silencing Effect and CCL22 Downstream Inhibition of ILsi

In order to determine the gene-silencing effect of ILsi, MC-38 cells were cultured on a 6-well plate at a density of 1 × 10^5^ cells/well and subsequently treated with the nanosystems containing different ILsi concentrations (5-50 nM) at 10 µg/mL X. ILsi@EM-Scr was also tested as a negative control. After 48 h, the cellular levels of IL messenger RNA (ILm) and protein were determined using quantitative real-time polymerase chain reaction (qRT-PCR) and western blotting analyses, respectively. The levels of secreted IL in the supernatant were detected using an ELISA kit. The silencing effects on MC-38 cells treated with the nanosystem, including ILsi-X, BP-H-X@EM, BP-H-ILsi-X@EM, and BP-H-ILsi-X@EM-Scr, were further evaluated using western blotting and ELISA analyses.

In order to investigate the effect of IL silencing on CCL22 secretion, macrophages (RAW264.7) and DCs derived from bone marrow suspensions were cocultured with MC-38 cells pretreated with the different configurations. After being cocultured in a transwell system (Corning, USA) for 48 h, the cell suspension was harvested to determine CCL22 levels using ELISA, whereas the levels of CCL22m in the macrophages or DCs were determined using qRT-PCR.

For western blotting, 50 µg of the cell lysate (per configuration) was first obtained by centrifuging MC-38 cells at 12000×*g* and 4^o^C for 20 min. The proteins were then separated using 10% SDS-PAGE and transferred to an Immobilon-P membrane (Millipore, USA). After incubating for 1 h in 5% skim milk dissolved in 1× TBST buffer (Thermo Fisher Scientific, USA), the membrane was washed three times (5 min per each washing) and incubated overnight in 5% BSA buffer (GE Healthcare Life Sciences, USA) supplemented with monoclonal IL antibody (1:1000; Santa Cruz Biotechnology, USA). The membrane was incubated for an additional 1 h with a second antibody (1:10000) before capturing the gel image using enhanced chemiluminescence substrates (Pierce™, Thermo Fisher Scientific, USA).

For qRT-PCR analysis, total RNA was obtained from MC-38 cells treated with TRIzol (Invitrogen, USA), and 2 µg of the resulting RNA was reverse-transcribed into cDNA using GoScript™ (Promega, USA). PCR was performed using SYBR Green (Thermo Fisher Scientific, USA), and all target-gene expression levels were normalized to the gene for mouse glyceraldehyde-3-phosphate dehydrogenase (gapdh). The murine primers used in the analyses were as follows: gapdh forward: 5′-CCACCCATGGCAAATTCCCATGGCA-3′. gapdh reverse: 5′-TCTAGACGGCAGGTCAGGTCCACC-3′. IL forward: 5′-AGTCGGCAAAGAAATCAAGATG-3′. IL reverse: 5′-CCTTGAAGGTGAAGTTGGACA-3′. CCL22 forward: 5′-CCTTCTTGCTGTGGCAATTCA-3′. CCL22 reverse: 5′-GGCAGCAGATACTGTCTTCCA-3′.

### Regulatory T (Treg) Cell Migration

Treg cells were isolated using a splenic single cell suspension from C57BL/6 mice using a magnetic sorting system (Miltenyi Biotec, Germany), and 3 × 10^5^ cells were then seeded on the upper chamber in a transwell plate (5.0 µm pore size; Corning, USA), whereas the cocultured supernatants of DCs and macrophages with pretreated MC-38 cells were introduced into the lower chamber. After incubating for 6 h, the cells in the lower chamber were collected and stained with PE-anti-mouse CD3, FITC-anti-mouse CD4, PerCP/Cy5.5-anti-mouse CD8, and APC-anti-mouse Foxp3 antibodies (Biolgend, USA) before counting using flow cytometry.

### Cell Cycle

MC-38 cells treated with the different configurations (containing 10 µg/mL X) and incubated for 24 h were harvested, washed twice with cold PBS, and kept in 95% ethanol at 0^o^C for 6 h. The cells were then washed twice with PBS and incubated with 1 mg/mL ribonuclease A for 15 min at room temperature, after which they were incubated with PI for the flow cytometry assay.

### ROS Generation, Apoptosis, and Live/Dead Assay

MC-38 cells (3 × 10^5^) were seeded on a 12-well plate and kept overnight. The configurations containing 10 µg/mL X and 50 nM ILsi were injected into the cells. For the NIR groups, the treated cells were exposed to an NIR laser (808 nm, 0.5 W/cm^2^, 5 min) after incubating for 6 h and then incubated again another 18 h. The cells were then collected and incubated with a 20 μM DCFDA probe for 30 min for the flow cytometry assay. Cell apoptosis resulting from ROS generation was also determined using the Annexin V/PI kit (BD Biosciences, USA). In addition, 6.7 μM AO and 750 μM PI were used for the live/dead assay.

### *In vitro* Cell Viability

MC-38 cells (2 × 10^4^) were seeded on a 96-well plate with 200 µL of cell culture medium and incubated overnight. The cells were treated with the configurations as a function of X concentration (0.1-50 µg/mL) at a fixed ILsi concentration (50 nM) and further incubated for 24 h. For the NIR groups, the treated cells were exposed to NIR irradiation (808 nm, 0.5 W/cm^2^, 5 min) after 6 h of incubation and then incubated again for 18 h. 10 µL of the MTT reagent (5 mg/mL) was added to each well for an additional 4 h of incubation. The supernatant in each well was removed and replaced with 100 µL of dimethyl sulfoxide to dissolve the formed formazan. Finally, a microreader (Thermo Fisher Scientific, USA) was used to measure the absorbance of each sample at a wavelength of 570 nm.

### Penetration of Tumor Spheroids

*In vitro* tumor spheroids were formed by seeding MC-38 cells (10^3^ cells/well) on 96-well plates with 2% low-melting-temperature agarose coating and incubated for 15 days (the medium was refreshed every two days). The spheroids were then transferred to six-well plates and treated with 50 nM BP-H-FAM-ILsi-X@EM in the absence and presence of YSA for 6 h. After washing twice with PBS, the fluorescence of the spheroids was observed using an Eclipse Ti fluorescent microscope (Nikon Instruments, USA).

### *In vivo* Biodistribution

For the analysis of biodistribution, BP-H-Cy5-ILsi-X@EM-YSA or BP-H-Cy5-ILsi-X@EM-Scr (set as 30 μg Cy5-ILsi per mouse, *N* = 6) was intravenously injected into MC-38 tumor-bearing mice having tumors ≥500 mm^3^. The fluorescence distributions in the mice were acquired at timepoints 0, 4, 8, 12, and 24 h using the Fluorescence-Labeled Organism Bioimaging Instrument (NeoScience, Republic of Korea). Corresponding *ex vivo* fluorescence distributions in the heart, liver, spleen, lung, kidney, and tumor were further examined by sacrificing the mice after 24 h.

### *In vivo* Antitumor Efficacy

The *in vivo* MC-38 tumor model was established by subcutaneous administration of 100 µL of MC-38 cells (1 × 10^6^) into the flank of six-week-old C57BL/6 mice. When the tumor size approached ~100 mm^3^, the mice were randomly divided into the six groups (*N* = 6) for tail-vein injections of the configurations containing fixed ILsi (30 µg per mice) and X (5 mg/kg). For the NIR groups, NIR (808 nm, 0.5 W/cm^2^, 5 min) was applied to the tumor sites 8 h after injection. Tumor sizes (volume [mm^3^] = the longest diameter [mm] × the shortest diameter^2^ [mm^2^]/2) were measured using a digital caliper, and the tumor weights were recorded at the end of the treatments.

In order to examine the *in vivo* silencing effect of ILsi, mice were first sacrificed 20 days after MC-38 tumor implantation. The tumors were harvested, homogenized, and lysed with M-PER^®^ (Thermo Fisher Scientific, USA) containing a 1% protease inhibitor. The resulting lysates were investigated using western blotting and IL ELISA (Biolegend, USA) kits. CCL22 levels in the tumors were also analyzed using a CCL22 ELISA kit (Sigma-Aldrich, USA). In order to assess immune system provocation, tumors were dissociated and homogenized as single cell suspensions in the presence of DNase I and collagenase IV. Following centrifugation using Percoll density gradient media (GE Healthcare Life Sciences, USA), the obtained lymphocytes were stained with surface markers CD4, CD8, B220, CD25, CD69, CD11c, and CD86, fixed and permeabilized with an Intracellular Fixation & Permeabilization Buffer Set (eBioscience, USA), and stained with Foxp3 antibody. The percentages of Treg, CD8^+^ T, CD11c^+^CD86^+^, and CD8^+^CD69^+^ cells were analyzed using flow cytometry. In order to determine IFN-*γ* and TNF-*α*, mouse serum was obtained from the treated mice and assessed using ELISA kits (Biolegend, USA). In addition, after sacrificing the mice, the hearts, livers, spleens, lungs, kidneys, and tumors were collected for H&E staining. The angiogenesis and proliferation of the tumors and intratumor CD8^+^ T and Treg cells were successively investigated by staining with markers CD31, Ki-67, CD8, and Foxp3.

All processes dealing with animal experiments were conducted in strict accordance with the guidelines and regulations of the Institutional Animal Ethical Committee of Yeungnam University, Republic of Korea.

### Statistical Analysis

All experimental data are displayed as the mean ± SD. One-way analysis of variance followed by Tukey's test was utilized to determine the differences among the treatment groups. Statistical significant was considered when* p* < 0.05.

## Results and discussion

**Figure 2:**
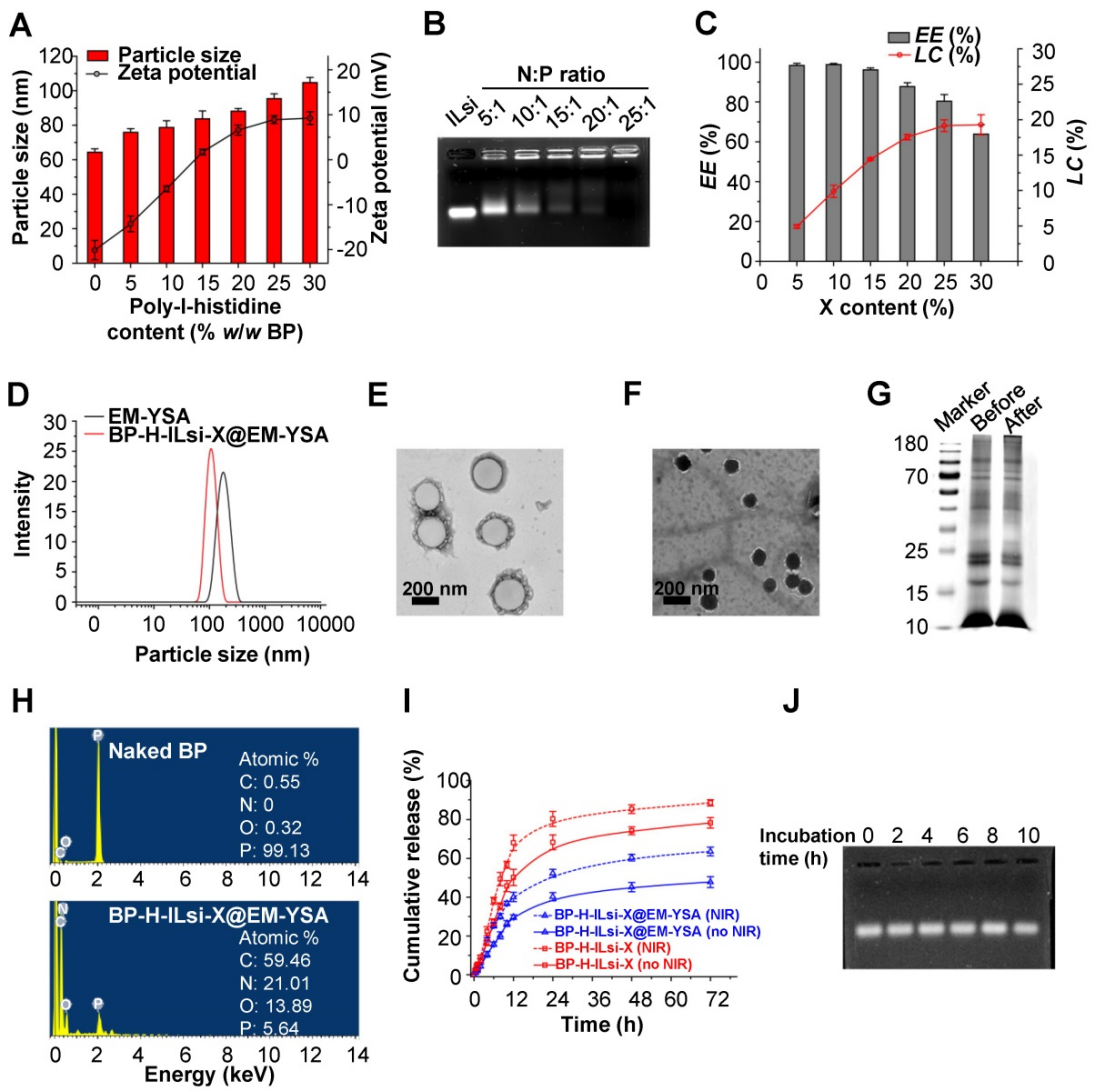
** Basic material characterization of BP-H-ILsi-X@EM-YSA nanosystems, including individual components.** (A) Changes in the DLS particle size and zeta potential as a function of H/BP fraction (*N* = 6). (B) Representative image of the gel retardation assay from different ILsi loading contents on BP-H (as different N/P ratios). (C) Changes in the *EE* and *LC* of the nanosystem as a function of X content (*N* = 3). (D-E) DLS size distributions and TEM images of the nanosystem, including individual EM-YSA. (G) Total protein levels of the nanosystem (noted as “after”), including individual EM-YSA (noted as “before”) confirmed using SDS-PAGE. (H) EDS profiles with elemental compositions of the nanosystem (bottom panel), including individual BP particles (top panel). (I) *In vitro* X release from the nanosystem, including BP-H-ILsi-X (for comparison), in the absence and presence of NIR irradiation at pH 6.5 (*N* = 6). The timepoints for NIR irradiation were set at 8, 12, and 24 h for 5 min (808 nm, 0.5 W/cm^2^). (J) Representative image of the ILsi state at 2, 4, 6, 8, and 10 h in the nanosystem after its incubation in mouse serum confirmed using agarose gel electrophoresis.

Changes in the size and zeta potential of dynamic light scattering (DLS) were examined by increasing the H content (0-30 *w*/*w*%) to graft to the surface of the tailored BP particles (forming BP-H). As shown in **Figure 2**A, the DLS size was proportional to the H content, and the >15% contents began to exhibit a positive potential for enabling ILsi loading on the BP-H through electrostatic binding. The stability of ILsi loading onto BP-H was evaluated using an agarose gel retardation assay (**Figure 2**B). The nitrogen/phosphate (N/P) ratio of 25:1 clearly exhibited complete ILsi retardation, which was represented by a decrease in the positive surface potential of BP-H from 12.14 to 6.08 mV (**Figure S2**), proving the strong electrostatic binding between BP-H and ILsi. The BP-H-ILsi-X@EM-YSA nanosystem was workable to load comparable amounts of si at the N/P ratio with the 80.8±3.5% encapsulation efficiency (*EE*) and 10.8±1.7% loading capacity (*LC*), as shown in **Figure S3**. The *EE* and *LC* of the BP-H-ILsi-X@EM-YSA nanosystem with different X contents were 87.6% and 19.15%, respectively (**Figure 2**C), which demonstrated the capacity of the nanosystem for high X loading. The DLS value of the EM-YSA prepared for cloaking was 368 nm, which was reduced to 174 nm after incorporation with BP-H-ILsi-X (**Figure 2**D). The coextrusion of BP-H-ILsi-X and EM-YSA through a 200 nm porosity membrane, as well as the flexibility of EM-YSA, might induce this size reduction (much smaller than native erythrocytes) to enhance the EPR effect and circulation. A representative transmission electron microscopy (TEM, after negative staining with uranyl acetate) image of EM-YSA (**Figure 2**E) matched the hydrodynamic size, whereas an image of the nanosystem (**Figure 2**F) showed particles significantly smaller than those of EM-YSA. The morphology of the nanosystem was more similar to that of the tailored BP particles than to that of EM-YSA, which proves that BP-H-ILsi-X was tightly cloaked with flexible EM-YSA in a core-shell construct from the extrusion. The formation of anchorable YSA was determined using Fourier transform infrared (FTIR) spectroscopy by comparing the spectra among the precursors (pristine YSA and 1,2-distearoyl-sn-glycero-3-phosphoethanolamine-N-carboxy[polyethylene glycol]-2000 [DSPE-PEG_2000_-COOH]) and product (YSA-PEG_2000_-DSPE), as shown in **Figure S4**A. The vibrational modes of CH in the PEG segment at ~2900 cm^-1^ and stretching vibrations from the amide bonding within the YSA peptide at between 1635 and 1660 cm^-1^ for YSA-PEG_2000_-DSPE supported the substitution reaction between the precursors to form anchorable YSA. The X-ray diffraction (XRD) spectra (**Figure S4**B) of naked BP and the nanosystem displayed only a difference in intensity of the characteristic BP peaks, implying that incorporation with H-ILsi-X and subsequent cloaking with EM-YSA did not significantly affect the BP crystallinity (exhibiting a workable stability). The disappearance of the characteristic H and X bands might represent tight EM-YSA cloaking of BP-H-ILsi-X to prevent H and X from escaping. The protein status of the base core@shell construct was confirmed using the sodium dodecyl sulfate-polyacrylamide gel electrophoresis (SDS-PAGE) protein assay (**Figure 2**G) and western blot analysis (**Figure S5**). The characteristic proteins of EM, CD47 and CD235a, were well preserved, and there were no significant differences between the base construct (only BP and EM) and the nanosystem (BP-H-ILsi-X@EM-YSA), which implies that the membrane protein was not significantly altered during nanosystem fabrication. EM cloaking was further examined using energy-dispersive X-ray spectroscopy (EDS) by comparing the spectra and elemental compositions of the naked BP and the nanosystem (**Figure 2**H). Significant increases in carbon, nitrogen, and oxygen (C, N, and O, respectively) after the formation of the nanosystem supported the existence of the membrane protein for the construction of the outer shell using EM. The generation of singlet oxygen (O=O, as potent reactive oxygen species [ROS]) in the nanosystem was explored by monitoring the absorbance of 1,3-diphenylisobenzofuran (DPBF, a probe for measuring O=O) in the presence of NIR irradiation (**Figure S6**A). The absorbance of DPBF decreased with increasing NIR exposure time, which implied an increase in O=O generation to dissociate the DPBF molecules (decreasing absorbance of DPBF). Reduction in the absorbance of the nanosystem was more rapid than that of naked BP, although BP is known as a catalyst to generate O=O under NIR irradiation 46. This might be the result of the protective function of EM at the surface region of the nanosystem, which suppresses undesirable fast bleaching of BP for maintaining oxygen generation 47. According to the results in **Figures S1**E and **S6**A, the designed core@shell construct might be suitable for creating not only mild hyperthermic effects, but also durable O=O generation. This EM function also affects the kinetics of drug release at pH 6.5 (simulated tumor microenvironment), as shown in **Figure 2**I. EM cloaking decreased the kinetics of X release (47.8% cumulative at 72 h) compared with that of noncloaked nanoparticles (78.8%), and this trend was identical in the presence of NIR irradiation (63.5% versus 88.0%), which suggests that the nanosystem enables sustainable and tumor-specific X release. The stabilities in dispersity and X release were further examined using different media (distilled water, phosphate-buffered saline [PBS], and mouse serum) for 10 h (**Figure S6**B). There were no notable rapid changes in the size and X content after the nanosystem (powder form) was dispersed, which exhibited suitability for injection. Agarose gel electrophoresis was used to visualize free ILsi from the nanosystem in order to determine its stability when incubated in mouse serum for 10 h (**Figure 2**J). No significant differences between incubation times represented the protective function of EM cloaking in maintaining ILsi activity.

**Figure 3:**
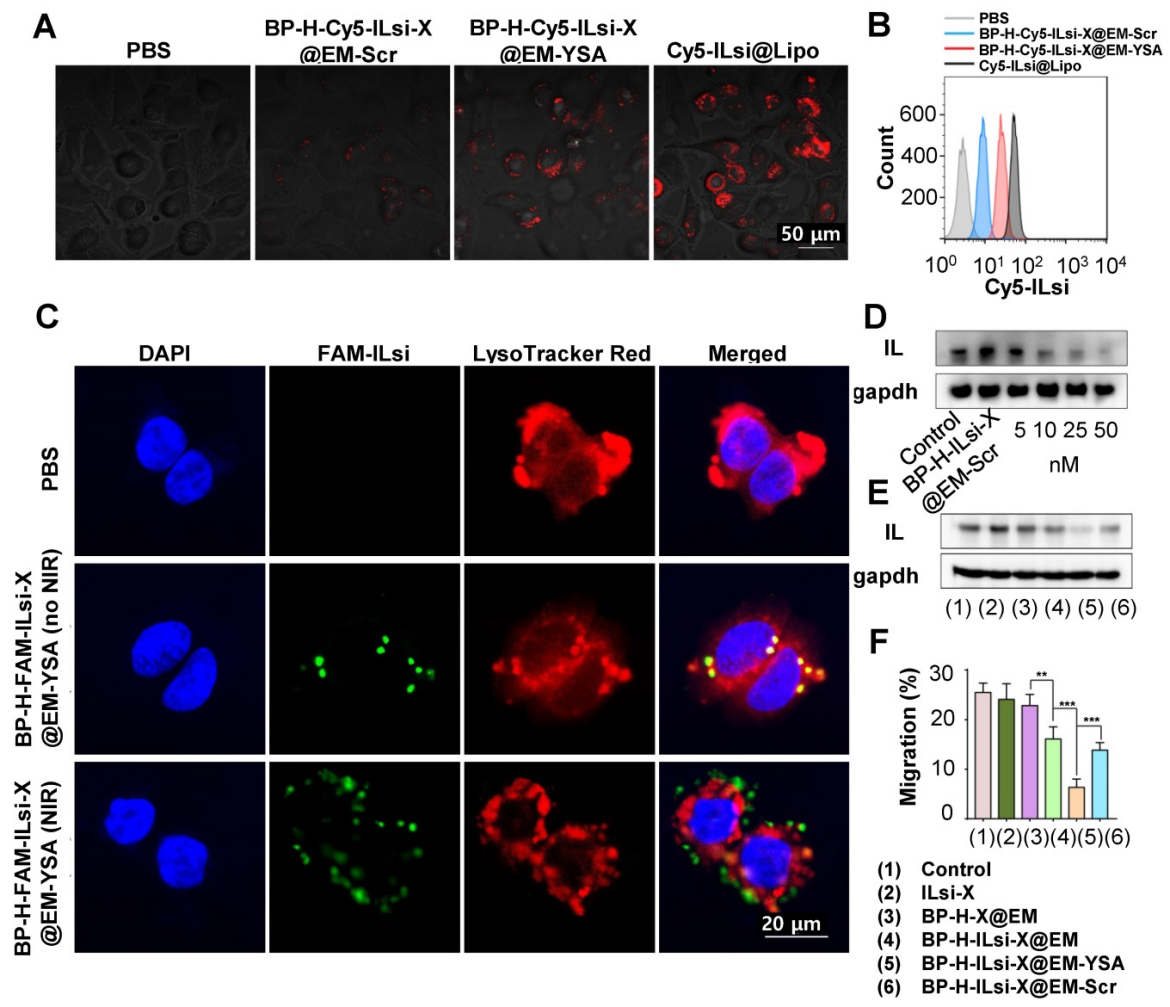
*** In vitro* study on the availability of BP-H-ILsi-X@EM-YSA (Cy5-ILsi or FAM-ILsi instead of ILsi) and BP-H-X-ILsi@EM-Scr (for a comparison purpose).** (A, B) Confocal images and flow cytometry data on MC-38 cells incubated with BP-H-Cy5-ILsi-X@EM-YSA for 6 h, including PBS, Cy5-ILsi@Lipo, and BP-H-Cy5-ILsi-X@EM-Scr, to evaluate cellular uptake levels. (C) Confocal analysis to examine the endosomal escape of ILsi from MC-38 cells after incubating with BP-H-FAM-ILsi-X@EM-YSA in the absence and presence of NIR irradiation (808 nm, 0.5 W/cm^2^, 5 min). DAPI and LysoTracker Red were used for nuclear and lysosome staining, respectively. (D) *In vitro* dose (ILsi)-dependent (5-50 nM) gene silencing effect on IL expression in MC-38 cells treated with the nanosystem, including ILsi@EM-Scr (for comparison). (E) Silencing effects of the different incubation configurations (1-6) with contents of 50 nM ILsi and 10 µg/mL X. (F) Percentage Treg cell migration after being cultured with the supernatant derived from the DC-macrophage-MC-38 coculture system (incubated with different configurations, 1-6, at fixed ILsi [50 nM] and X [10 µg/mL] contents [*N* = 6;^ **^p < 0.01;^ ***^p < 0.001]).

The intracellular uptake of a cyanine 5 (Cy5)-labeled nanosystem (*i.e.*, BP-H-Cy5-ILsi-X@EM-YSA) was visualized using confocal laser scanning microscopy (CLSM) and compared with scrambled EM (EM-Scr) and commercial lipofectamine (Lipo) cloaked configurations (*i.e.*, BP-H-Cy5-ILsi-X@EM-Scr and Cy5-ILsi@Lipo). The fluorescence intensity of the Cy5-labeled nanosystem was comparable to that of EM-Scr, whereas MC-38 cells treated with BP-H-Cy5-ILsi-X@EM-Scr did not produce distinguishable fluorescence signals because of the absence of YSA (**Figure 3**A). The quantitative levels from the different configurations that were determined using flow cytometry matched the CLSM observations, which prove YSA's targetability to cancer cells (**Figure 3**B). CLSM was also used to analyze the endosomal escape of ILsi (a vital role in gene silencing) after cells were incubated with a fluorescein amidite (FAM) labeled nanosystem in the absence and presence of NIR irradiation (**Figure 3**C). By merging FAM-ILsi (green) with 4′,6-diamidino-2-phenylindole (DAPI, blue) and LysoTracker Red signals, the dislocation of green dots (FAM-ILsi) from the lysosome was clearly confirmed in the presence of NIR irradiation, whereas FAM-ILsi remaining in the lysosome was observed as yellow (green + red) dots in the absence of NIR irradiation, elucidating another function of the nanosystem for NIR-triggered endosomal escape of ILsi. The NIR-triggered escape of uptaken nanosystems has been introduced in a previous report, where the produced ROS (mainly singlet oxygen), oxidized the outer cover that leads release of the therapeutic components for interacting with their targets in the cytosol 48. Western blotting images from MC-38 cells treated with the nanosystem containing different amounts of ILsi (5-50 nM) exhibited a clearer dose-dependent attenuation of IL expression than BP-H-ILsi-X@EM-Scr (**Figure 3**D). Flow cytometry (**Figure S7**A) and qRT-PCR (**Figure S7**B) analyses were also conducted to, respectively, determine dose-dependent downregulated IL levels and ILm expressions in the cells treated with the nanosystem containing different amounts of ILsi (5-50 nM), further proving the critical role of YSA. Attenuation in IL (**Figure 3**E) and ILm (**Figure S7**C) expressions was also evaluated for the different configurations (1-6). IL and ILm expression was significantly attenuated (silencing effect) in ILsi-loaded base (BP@EM) constructs (4-6) compared to that in the other constructs. In particular, nanosystem (5) exhibited the greatest silencing effect resulting from YSA's targetability, which ensured the highest uptake into cancer cells for inhibiting IL and ILm expressions. Because the C-C motif chemokine 22 (CCL22, mainly produced by macrophages and dendritic cells [DCs] through stimulation of cancer-cell-derived IL) 44,49 expressions are commonly found in various tumor tissues and are critical for the recruitment of Treg cells to tumor tissues 50, the MC-38 cells (treated with 1-6) were cocultured (simulating the tumor microenvironment) with RAW264.7 macrophages (derived from splenocytes) or bone marrow DCs (BMDCs) to estimate the levels of CCL22 and CCL22m using enzyme-linked immunosorbent assay (ELISA) and qRT-PCR analysis, respectively. As shown in **Figure S7**D, the nanosystem exhibited the greatest effect on inhibiting CCL22 secretion (from IL silencing) in the macrophages cocultured with the MC-38 cells treated with 1-6 compared to those of other configurations. In this assay, YSA also played an important role in the downregulation of CCL22 compared with that of nontargeted EM groups (4 and 6), which matched the CCL22m expressions from the treatments (1-6) that were determined through qRT-PCR analysis (**Figure S7**E). In the same experiments for coculturing with BMDCs, analogous trends in CCL22 (**Figure S7**F, protein level) and CCL22m (**Figure S7**G, m levels) expressions appeared (*i.e.*, reducing tumor cell-derived IL levels inhibits CCL-22 secretion in the macrophages and DCs), although the scales of CCL22 and CCL22m were different from those of the macrophages. In particular, significantly greater levels of CCL22 secretion were observed in the DCs than in the macrophages, because DCs are the major source of CCL22 in mice (and humans) 51. Nanosystem (5) treatment exhibited the lowest migration of Treg cells (effectively inhibiting the migration behavior) (**Figure 3**F), which further confirms that the architecture of the nanosystem allows the modulation of IL-induced CCL22 secretion to impeded Treg cell migration.

**Figure 4:**
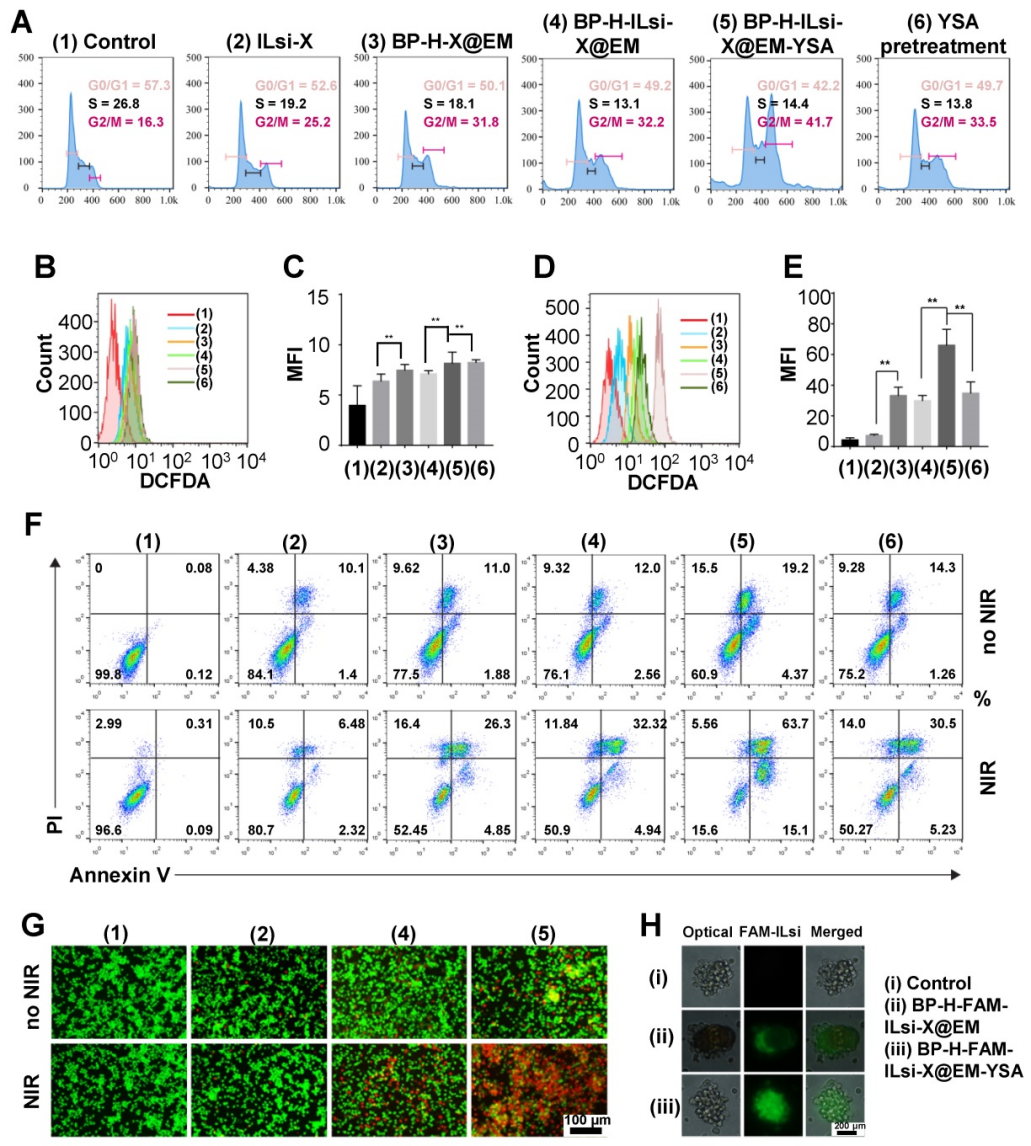
***In vitro* study on the functionality of the nanosystem, including other configurations (1-6).** Configuration 6 represents the nanosystem treatment after pretreatment with YSA. (A) Cell-cycle analysis of MC-38 cells treated with the different configurations and containing 50 nM ILsi and 10 µg/mL X. (B-E) ROS generation and MFI (corresponding statistical ROS) levels in MC-38 cells treated with the different configurations (1-6) in the absence (B, C) and presence (D, E) of NIR irradiation (808 nm, 0.5 W/cm^2^, 5 min, ^**^p < 0.01). (F, G) Apoptosis and live/dead assays of MC-38 cells incubated with the different configurations in the absence and presence of irradiation. (H) Tumor spheroid models to examine the penetration of BP-H-ILsi-X@EM in the absence of presence of YSA.

Flow cytometry was used to analyze the cell cycles of the treated MC-38 cells treated with 1-6 (**Figure 4**A). The fraction of the G2/M phase for ILsi-X (2) significantly increased after being loaded onto BP-H and subsequent EM cloaking (4), and was further enhanced by YSA anchoring (5) because of its targetability to the MC-38 cells. On the other hand, pretreatment (saturation) of the cells with YSA before treating them with nanosystem (6) induced similar G2/M phase arrest with BP-H-ILsi-X@EM (4), which represents the importance of YSA. ROS generation (in terms of flow cytometry results and mean fluorescence intensity [MFI]) from the MC-38 cells treated with 1-6 in the absence (**Figures 4**B and **4**C) and presence (**Figures 4**D and **4**E) of NIR irradiation was assessed using 2′,7′-dichlorodihydrofluorescein diacetate (DCFDA) cellular ROS detection assay. The levels of ROS in treatments (3-6) significantly increased after NIR irradiation. In particular, nanosystem (5) exhibited the highest activity for ROS generation (approximately two times greater than treatment with 3, 4, or 6). This implies that the targetability of the nanosystem enhanced by anchored YSA can amplify the photodynamic effects after NIR irradiation. As an aftereffect of cell-cycle arrest, apoptosis of MC-38 cells was determined using the Annexin V/propidium iodide (PI) kit in the absence and presence of NIR irradiation (**Figure 4**F). Increases in the populations of late apoptotic cells in treatments (3-6) after NIR irradiation greatly coincided with those from the ROS detection assay (**Figures 4**B-**4**E). From acridine orange (AO)/PI staining, the dead cells from treatments (1, 2, 4, and 5) could be visualized in the absence and presence of NIR irradiation (**Figure 4**G), and many more were found in treatment (5) than in other treatments. The cell viabilities on MC-38 (**Figure S8**A) and 293T (**Figure S8**B) determined using 3-(4,5-dimethylthiazol-2-yl)-2,5-diphenyltetrazolium bromide (MTT) assay for the treatments with and without NIR also matched the results of the ROS and apoptosis assays. Interestingly, EM-YSA incorporated BP nanosystem did not exhibit significant cytotoxicities on normal kidney (293T) cells (**Figure S8**B) excluding free X, which may be because of low expression of YSA receptor on 293T cells that induces lower cellular internalization of the nanosystem. In addition, there were slight differences in hemolysis (**Figure S9**) were observed between the nonclassified and classified BP (including BP [classified]-H-ILsi-X@EM-YSA) at high concentrations (>200 μg/mL), although no significant hemotoxicities were detected at all concentrations, suggesting that morphological uniformity of BP is also valid to strongly suppress the generation of toxic effects. Tumor spheroid penetration was further observed in BP-H-FAM-ILsi-X@EM and BP-H-FAM-ILsi-X@EM-YSA configurations using fluorescence microscopy (**Figure 4**H). Green FAM-ILsi dots were distributed only at the outside corners in the absence of YSA, whereas they were distributed most inside the spheroid in the configurations with YSA, proving that anchoring YSA promotes the penetration of the nanosystem into the depths of the tumor tissues.

**Figure 5:**
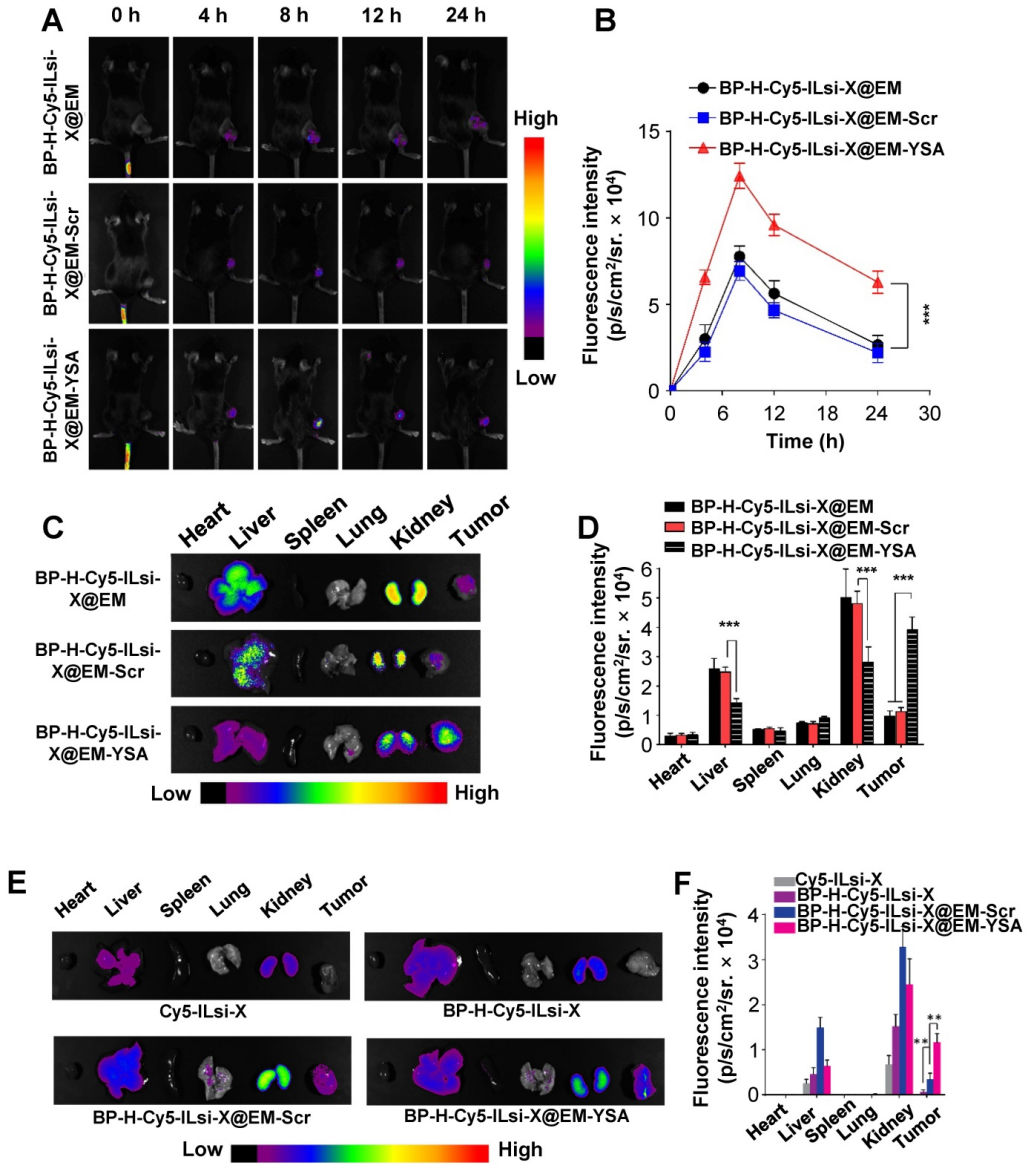
** Biodistribution of BP-H-ILsi-X@EM in the absence and presence of YSA in MC-38 tumor-bearing mice.** (A, B) Representative fluorescence contours and corresponding intensities captured at different timepoints after intravenous injection of BP-H-Cy5-ILsi-X@EM, BP-H-Cy5-ILsi-X@EM-Scr, BP-H-Cy5-ILsi-X@EM-YSA (*N* = 6; ^***^p < 0.001). (C, D) *Ex vivo* fluorescence contours and corresponding intensities for major organs and tumor 24 h after injection (*N* = 6; ^***^p < 0.001). (E, F) *Ex vivo* fluorescence contours and corresponding intensities for major organs and tumor 48 h after injection (*N* = 6; ^**^p < 0.01). Cy5-ILsi-X and BP-H-Cy5-ILsi-X were tested as non-EM-cloaked configurations for comparison.

In order to observe the biodistribution of the nanosystem, MC-38 tumor-bearing mice were intravenously injected with either BP-H-Cy5-ILsi-X@EM, BP-H-Cy5-ILsi-X@EM-YSA, or BP-H-Cy5-ILsi-X@EM-Scr for fluorescence imaging. An accumulation of Cy5-ILsi in the tumor site was observed 4 h after injection in both configurations, and fluorescence was maximized 8 h after injection (**Figure 5**A). The quantified fluorescence results (**Figure 5**B) showed a difference (approximately two times) between the configurations, demonstrating the critical role of YSA in tumor-specific accumulation, even *in vivo*. After sacrificing the mice 24 h posttreatment, *ex vivo* fluorescence images (**Figure 5**C) and levels (**Figure 5**D) in the major organs and tumors were obtained. Even though fluorescence was also detected in other organs (kidney and liver) because of the size distribution of nanosystem (<200 nm), biodegradation of the classified BP was approximately 4 mass percent per day, suggesting suitability for ensuring biodegradability. The significantly greater tumor-specific accumulation of BP-H-Cy5-ILsi-X@EM-YSA was consistent with the results shown in **Figures 5**A and **5**B, which supports the enhanced tumor accumulation effect resulting from YSA anchoring. The differences in temperature elevation profile matched the greater temperature increases from BP-H-ILsi-X@EM-YSA treatment (**Figure S10**) in a gentle manner (no more than 4^o^C), while negligible temperature elevation was observed from PBS treatment. Extended monitoring (48 h) of fluorescence intensities (**Figures 5**E and **5**F) exhibited a similar trend in the results of biodistribution seen 24 h posttreatment and confirmed the importance of EM cloaking (deriving erythrocyte mimicry) by additionally comparing the results with Cy5-ILsi-X and BP-H-Cy5-ILsi-X, which displayed nearly no fluorescence signals in tumors 48 h after injection.

**Figure 6:**
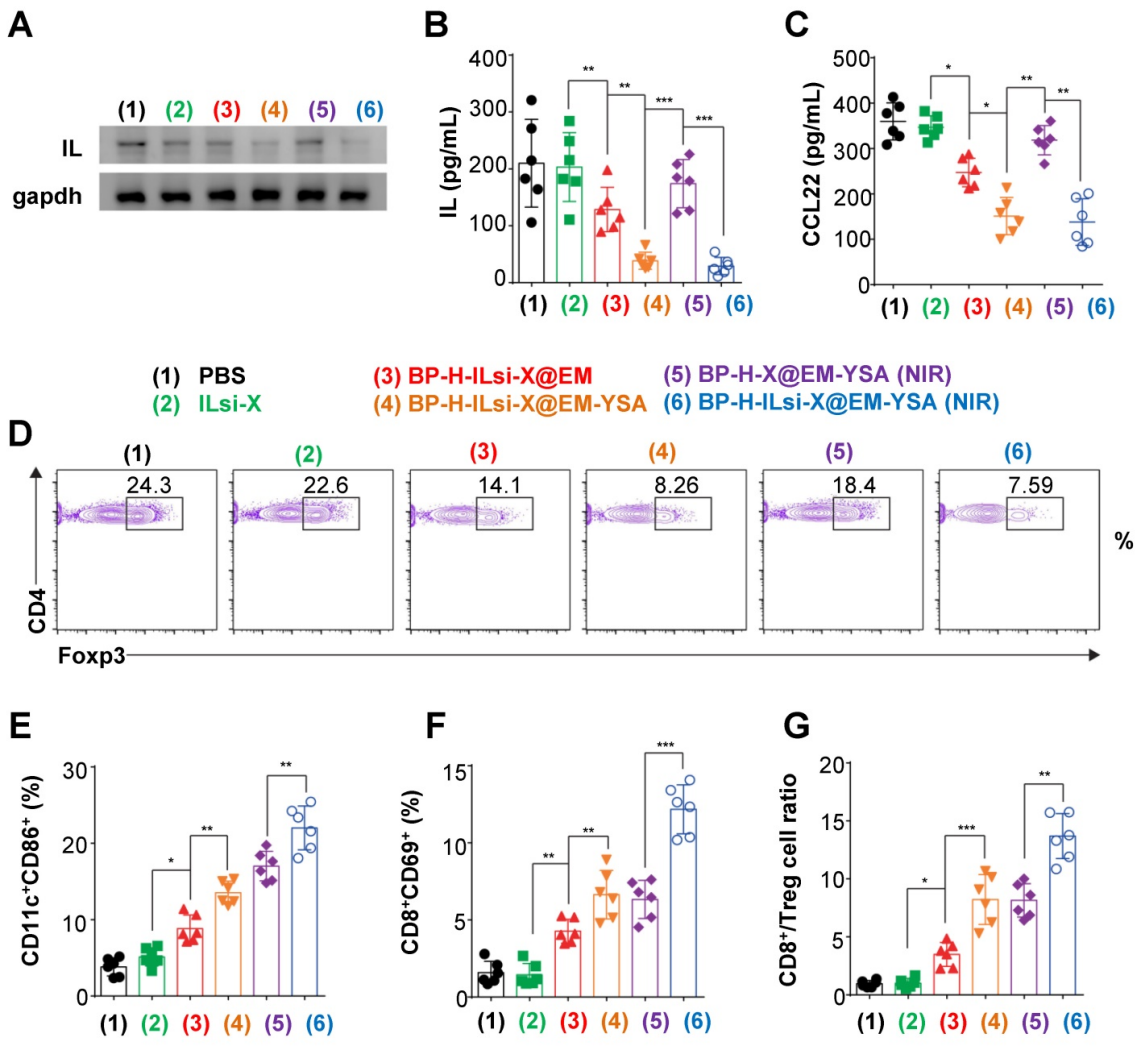
*** In vivo* assessment of the abrogation of Treg cell infiltration to examine antitumor responses from the nanosystem.** (A, B) Western blotting and ELISA to determine IL levels in MC-38 tumors treated with the different configurations (1-6) (*N* = 6). (C) ELISA analysis to measure intratumoral CCL22 levels after treatments with the configurations (1-6). (D) Flow cytometry analysis to identify intratumoral Treg cell contents in the treated mice (1-6). (E-G) Flow cytometry analysis to estimate the percentages of CD11c^+^CD86^+^ (dendritic) and CD8^+^CD69^+^ (T) cells and ratio between CD8^+^ and Treg in the treated tumors (1-6). (*N* = 6; ^*^p < 0.05,^ **^p < 0.01;^ ***^p < 0.001)

In order to determine the *in vivo* antitumor effect, MC-38 cell-bearing C57BL/6 mice were intravenously injected with the nanosystem, including other configurations. According to western blot analysis (**Figure 6**A), there were no robust effects on IL silencing within the tumor sites treated with the configurations that excluded ILsi or YSA (2 [because of no vehicles, protecting layers, and targeting agents], 3, and 5), whereas IL expression significantly decreased in configurations containing both ILsi and YSA (4 and 6), and this result was further enhanced in the presence of NIR irradiation (6). ELISA measurements of IL (**Figure 6**B) and CCL22 (**Figure 6**C) levels after treatments (1-6) were consistent with the results of western blotting, proving the importance of YSA (enabling tumor targeting) in silencing IL and subsequently inhibiting CCL22 secretion, even *in vivo*. The levels of tumor-associated Treg cells also matched the levels of IL and CCL22, which was verified by measuring forkhead box P3 (Foxp3, Treg cell lineage specification factor) (**Figure 6**D). In particular, the nanosystem with NIR irradiation (6) exhibited the strongest suppression of Treg cell accumulation in the tumor microenvironment, implying that effective silencing of IL levels in tumors can successively and significantly inhibit CCL22 secretion and Treg cell accumulation, demonstrating an antitumor activity. Treg cell accumulation is also critical for inhibiting DC maturation, as well as for disturbing CD8^+^ T cell accumulation and activation 52. In order to confirm the aftereffect of inhibiting Treg cell accumulation, the levels of CD11c^+^CD86^+^ (DC maturation marker, **Figure 6**E) and CD8^+^CD69^+^ (T cell activation marker, **Figure 6**F) were estimated after treatments (1-6). For CD11c^+^CD86^+^, the configurations that included NIR irradiation (5 and 6) exhibited higher levels of DC maturation, most likely because of its NIR-triggered enhanced secretion in the tumor 53. Due to the fact that mature DCs have the capacity to present tumor-associated specific antigens to CD8^+^ T cells 54, ensuring the highest levels of mature DCs from the nanosystem with NIR irradiation might be effective for the activation and proliferation of T cells capable of recognizing tumor cells. The nanosystem with NIR irradiation (6) also derived the highest levels of CD8^+^CD69^+^, including the ratio of CD8^+^ T to Treg cells in the tumors (**Figure 6**G), and the trend derived from treatments (1-6) was inversely proportional to IL silencing levels, CCL22 secretion, and Foxp3. Configuration 6 further induced the highest levels, even with interferon-gamma (IFN-*γ*, a pleiotropic cytokine produced by T cells, **Figure S11**A) and tumor necrosis factor-alpha (TNF-*α*, a proinflammatory cytokine, **Figure S11**B), which suggests that an effective combination among ILsi, YSA, and NIR irradiation can elicit favorable immunological outcomes against tumor growth.

**Figure 7:**
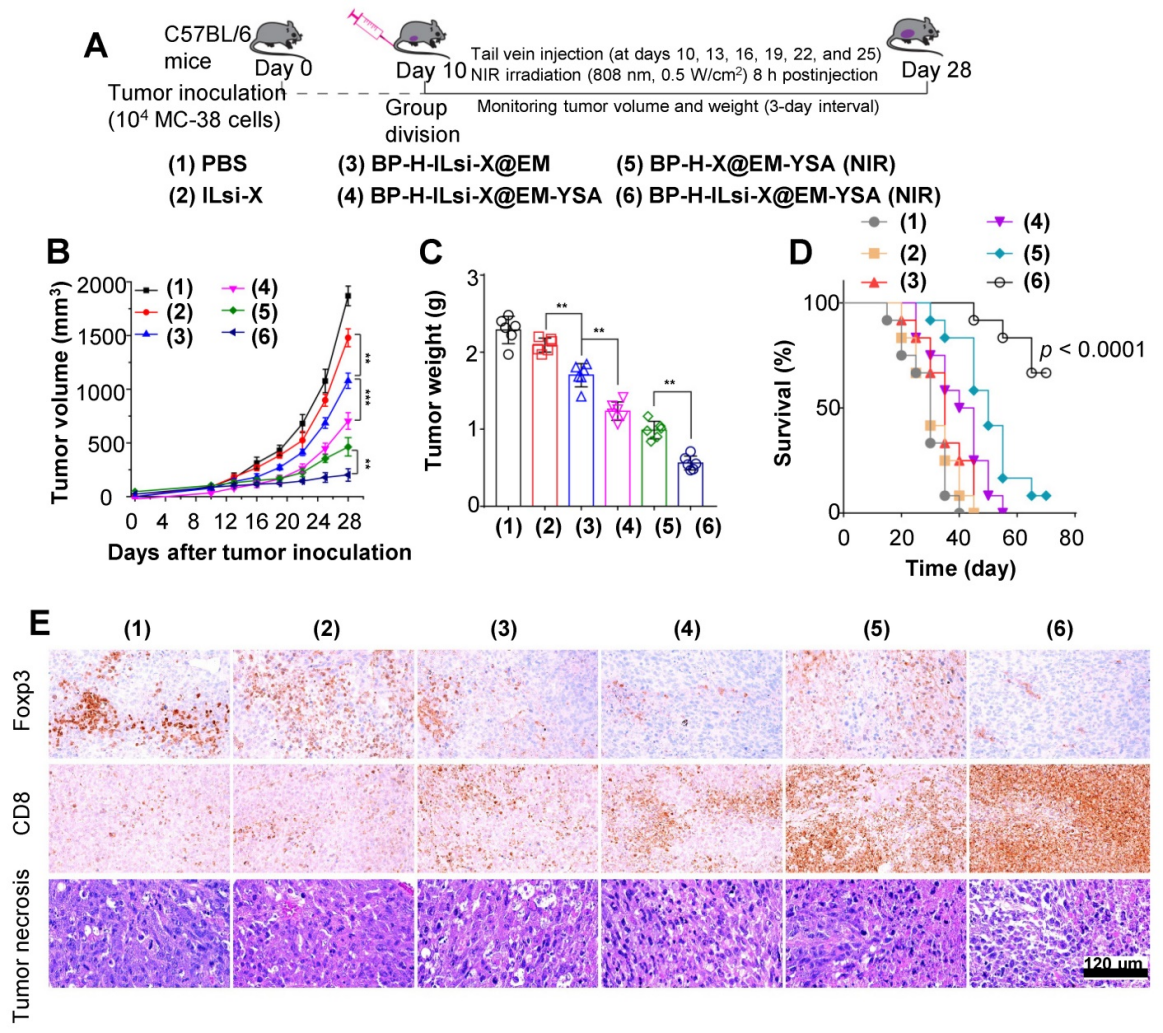
*** In vivo* antitumor effects of the nanosystem against MC-38 tumor model.** (A) A schematic of the procedure to evaluate *in vivo* antitumor effects. MC-38 cells were subcutaneously injected into the flank of C57BL/6 mice. Mice were randomly divided into groups when their tumors reached 100 mm^3^, and the mice were treated with the different configurations (1-6) through an injection into the tail vein at the indicated time intervals. (B, C) Monitored tumor volume of the treated mice during the period and the final tumor weight extracted from the sacrificed mice (^**^p < 0.01;^ ***^p < 0.001). (D) Survival curve of mice treated with the configurations (1-6) (*N* = 12 in each group). (E) Histopathological and immunohistochemical levels of intratumoral Foxp3 Treg and CD8^+^ T cells from representative tumor sections of the treated mice.

The tumor size and survival rate were monitored to assess systemic effect of treatments (1-6), including conducting histopathological and immunohistochemical analyses of tumor mass, as depicted in **Figure 7**A. The suppression of tumor growth for the nanosystem with NIR irradiation (6) was greater than that of other configurations (1-5), as shown in **Figures 7**B, **7**C, and **S12**, which once again proves the effectiveness of a combination of ILsi, YSA, and NIR irradiation. The survival analysis exhibited a significant difference between the nanosystem with NIR irradiation and the others (**Figure 7**D), collectively demonstrating a better therapeutic outcome from effective combinations of ILsi, YSA, and NIR irradiation. The lowest (or highest) level of Foxp3 (or CD8) was observed within the tumor area (*i.e.*, exhibiting the lowest Treg cell frequency while attracting the highest number of CD8^+^ T cells) with notable necrosis after treatment with the nanosystem in the presence of NIR irradiation (**Figure 7**E and **Table S1**). The observed Ki-67 (a tumor proliferation marker) and CD31 (an angiogenesis marker) levels further support the outstanding features of the nanosystem with NIR irradiation for suppressing the proliferation of tumor cells and angiogenesis of tumor vessels (**Figure S13** and **Table S1**). Hematoxylin and eosin (H&E) staining of the major organs elucidated that the nanosystem did not result in notable toxicities even with NIR irradiation, which warrants biosafety for further clinical applications (**Figure S14**).

## Conclusions

Coarse BP flakes were in-flight-tailored by electrostatically classifying (as 60 nm) pulverized BP aerosol particles using a Boltzmann charge distribution. The tailored BP particles were more biocompatible than untailored BP particles because of the reduction in the morphological irregularity, and EM cloaking further enhanced that biocompatibility and derived mild photothermal effects under NIR irradiation. These preferential properties motivated us to fabricate <200 nm BP-H-ILsi-X@EM-YSA core@shell nanosystems (EPR-enabling) by loading ILsi and X on H-grafted BP (BP-H) as the inner core while anchoring YSA on EM as the outer shell for enhancing targeted, durable, and mild cancer chemophotoimmunotherapy. EM cloaking suppressed undesirable rapid X release, and anchoring YSA and NIR irradiation significantly enhanced tumor targetability and endosomal escape to maximize the silencing effect of ILsi. The nanosystem abrogated IL expression, shut down after CCL22 secretion, and restricted Treg cell accumulation within the tumor site to induce sufficient antitumor immune responses (*i.e.*, activated sufficient CD8^+^ T cells, IFN-*γ*, and TNF-*α*) with X- and NIR-induced therapeutic effects. Taken together, the in-flight tailoring of BP particles provides a promising base core for fabricating <200 nm EM-mimicking multifunctional nanosystems, which could be beneficial for constructing smarter nanoarchitectures to use in combination cancer therapies.

## Supplementary Material

Supplementary figures and tables.

## Abbreviations

EPR: enhanced permeability and retention
NIR: near-infrared
BP: black phosphorus
EM: erythrocytes membrane
YSA: ephrin-A2 receptor-specific peptide
H: poly-L-histidine
ILsi: interleukin-1*α* silencing small interfering RNA
X: paclitaxel
GMD: geometric mean diameter
GSD: geometric standard deviation
NDMA: nanodifferential mobility analyzer
HPLC: high-performance liquid chromatography
DLS: dynamic light scattering
EE: encapsulation efficiency
LC: loading capacity
TEM: transmission electron microscopy
FTIR: Fourier transform infrared
XRD: X-ray diffraction
SDS-PAGE: sodium dodecyl sulfate-polyacrylamide gel electrophoresis
EDS: energy-dispersive X-ray spectroscopy
DPBF: 1,3-diphenylisobenzofuran
PBS: phosphate-buffered saline
Cy5: cyanine 5
CLSM: confocal laser scanning microscopy
FAM: fluorescein amidite
DAPI: 4′,6-diamidino-2-phenylindole
qRT-PCR: quantitative real-time polymerase chain reaction
CCL: C-C motif chemokine
T: Treg
BMDC: bone marrow dendritic cell
ELISA: enzyme-linked immunosorbent assay
AO: acridine orange
MTT: 3-(4,5-dimethylthiazol-2-yl)-2,5-diphenyltetrazolium bromide
Foxp3: forkhead box P3
IFN-*γ*: interferon-gamma
TNF-*α*: tumor necrosis factor-alpha
H&E: Hematoxylin and eosin
