# Supplementary material for: Tailored Black Phosphorus for Erythrocyte Membrane Nanocloaking with Interleukin-1α siRNA and Paclitaxel for Targeted, Durable, and Mild Combination Cancer Therapy
Source: Theranostics. 2019 Sep 19;9(23):6780–96. doi: 10.7150/thno.37123 (PMC6815959; doi:10.7150/thno.37123)
Supplement: Supplementary file 1 — Supplementary figures and tables. [file thnov09p6780s1.pdf]

Supplementary material for

Tailored Black Phosphorus for Erythrocyte Membrane Nanocloaking with Interleukin-1α siRNA and Paclitaxel for Targeted, Durable, and Mild Combination Cancer Therapy

Wenquan Ou[1], Jeong Hoon Byeon[2*], Zar Chi Soe[1], Bo Kyun Kim[1], Raj Kumar Thapa[1], Biki Gupta[1], Bijay Kumar Poudel[1], Sae Kwang Ku[3], Chul Soon Yong[1], Jong Oh Kim[1*]

Mr. Wenquan Ou, Mr. Zar Chi Soe, Mr. Bo Kyun Kim, Mr. Raj Kumar Thapa, Dr. Biki Gupta, Dr. Bijay Kumar Poudel, Dr. Chul Soon Yong, and Dr. Jong Oh Kim

1. College of Pharmacy, Yeungnam University, Gyeongsan 38541, Republic of Korea.

Dr. Jeong Hoon Byeon

2. School of Mechanical Engineering, Yeungnam University, Gyeongsan 38541, Republic of Korea.

Dr. Sae Kwang Ku

3. College of Korean Medicine, Daegu Haany University, Gyeongsan 38610, Republic of Korea.

[*]Corresponding authors: J. H. Byeon. Email: postjb@yu.ac.kr and J. O. Kim. Email: jongohkim@yu.ac.kr

Figure S1

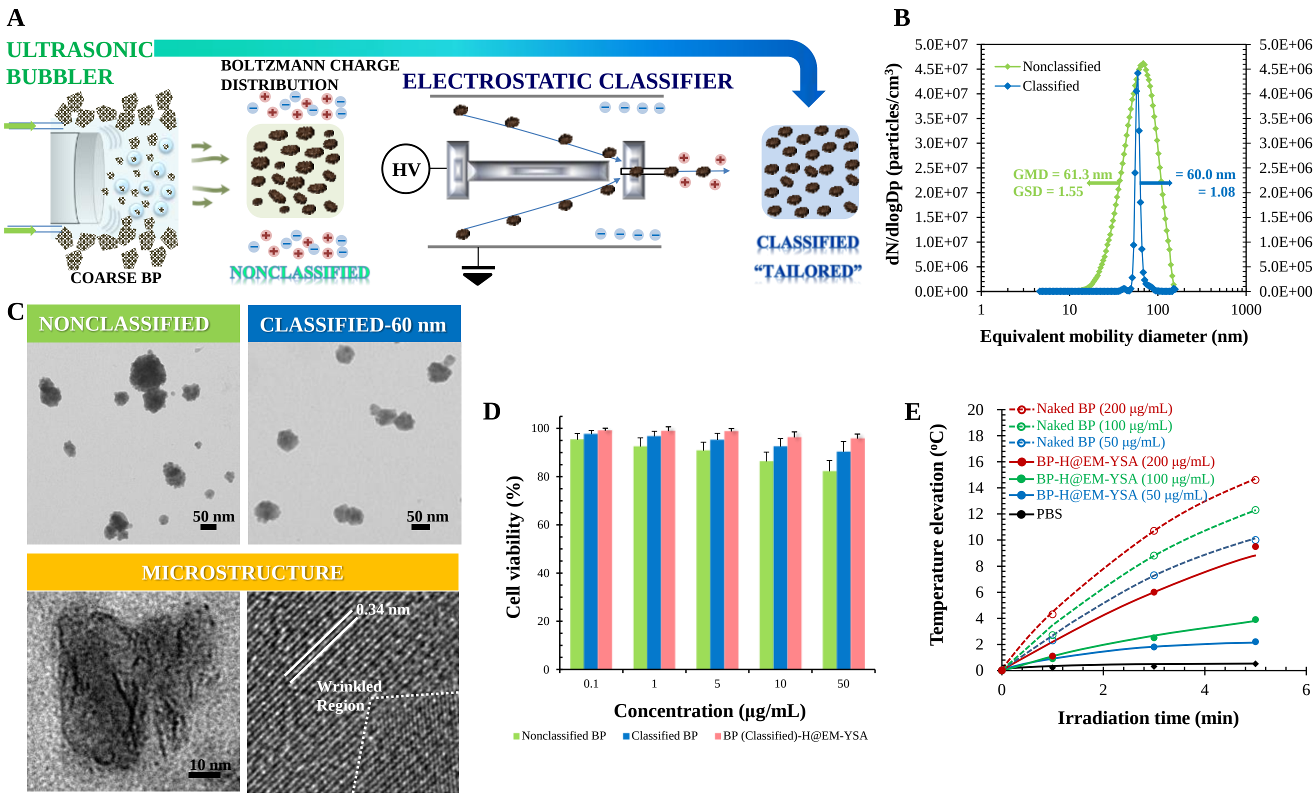

In-flight tailoring of coarse BP flakes and basic material characterizations. (A) A schematic of in-flight tailoring for the continuous production of size-classified (tailored) BP particles. Coarse BP flakes were first pulverized using a probe sonicator after being dispersed in deoxygenated water (1 mg/mL). The dispersion was mechanically sprayed as droplets with nitrogen gas, and the droplet-laden nitrogen gas flow entered a diffusion dryer to form BP aerosol particles by absorbing water molecules. The aerosols were then injected into a NDMA after passing through a soft X-ray charger (creating Boltzmann charge distribution) for a size classification of 60 nm. (B) Size distributions of untailored (nonclassified; GMD = 61.3 nm, GSD = 1.55) and tailored (classified; GMD = 60.0 nm, GSD = 1.08) BP particles. (C) TEM images of the BP particles. The classified configuration exhibited a more uniform distribution than that of the nonclassified configuration. The high-magnification images show the wrinkled shape and characteristic microstructure of BP (021 plane). (D, E) Concentration-dependent cytotoxicities (MC-38 cells) and NIR-activated (808 nm, 0.5 W/cm^2, 5 min) temperature elevations of the tailored (naked) and EM-cloaked BP specimens. The tailored BP exhibited better biocompatibility than that of the untailored BP, and this was further enhanced by EM cloaking. Cloaking restrained the temperature elevations of naked BP particles ($\Delta T \leq 9^{o}$C) to enhance the mild hyperthermic microenvironment.

Figure S2

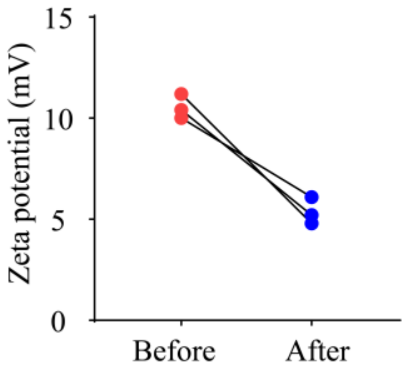

Zeta potential changes of BP-H (H-grafted BP) particles after ILsi loading (N = 3). The decrease in the positive potential of BP-H could be the result of ILsi's negative charge, representing successful loading of ILsi on BP-H.

Figure S3

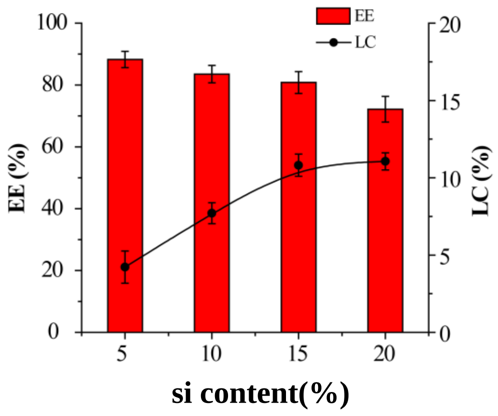

Changes in *EE* and *LC* of the nanosystem as a function of siRNA content (N = 3).

Figure S4

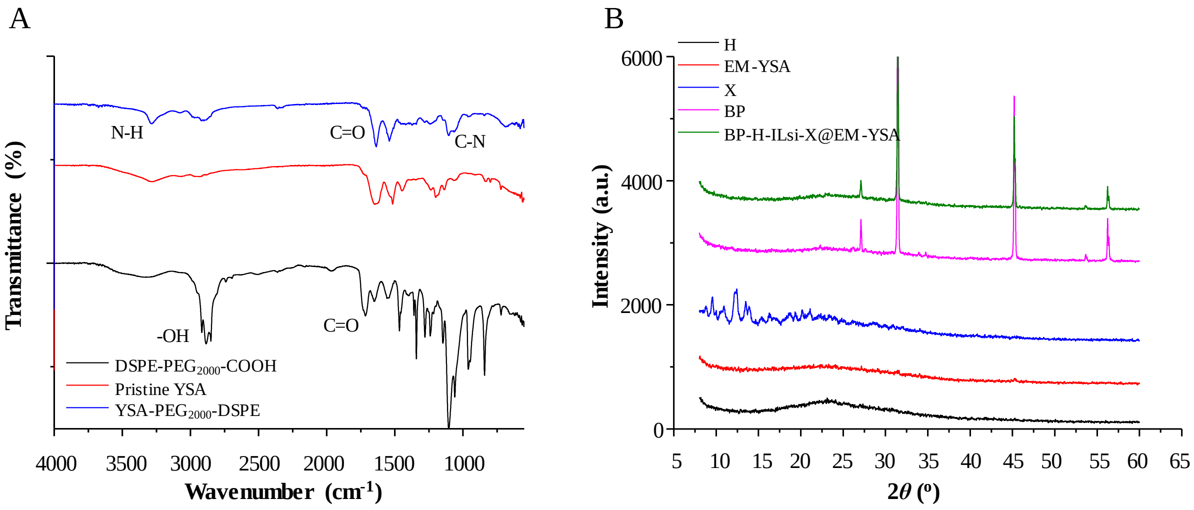

FTIR spectrum of anchorable YSA (YSA-PEG$_{2000}$-DSPE) (A) and XRD profile of BP-H-ILsi-X@EM-YSA (B) with spectra of individual components to verify the loading of functional molecules on core BP particles.

Figure S5

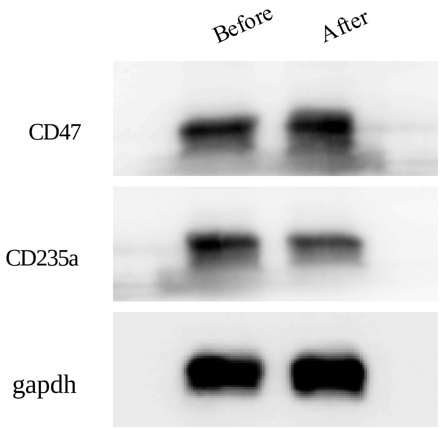

Western blot analyses of CD47 and CD235a proteins in EMs before and after the assembly of BP-H-ILsi-X@EM-YSA.

Figure S6

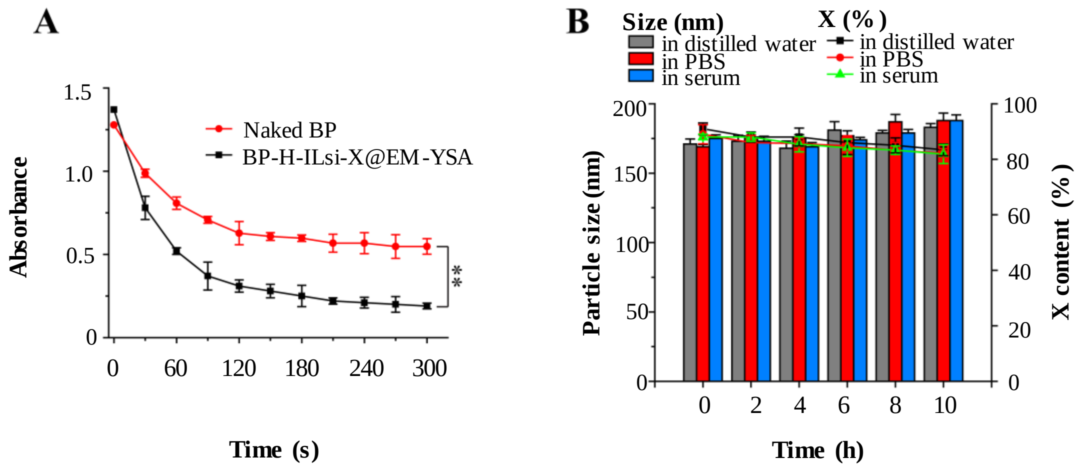

(A) Absorbance monitoring of DPBF at 410 nm for 5 min NIR irradiation (808 nm, 0.5 W/cm^2) after treatment with naked BP particles and BP-H-ILsi-X@EM-YSA nanosystems to examine the generation of singlet oxygen (as potent ROS generation). The concentration of BP for this monitoring was 100 μg/mL (N = 6). (B) Monitoring of the DLS size and X content of the nanosystem for 10 h after being dispersed in distilled water, PBS, and mouse serum to examine dispersion stability (N = 6; $^{**}p < 0.01$).

Figure S7

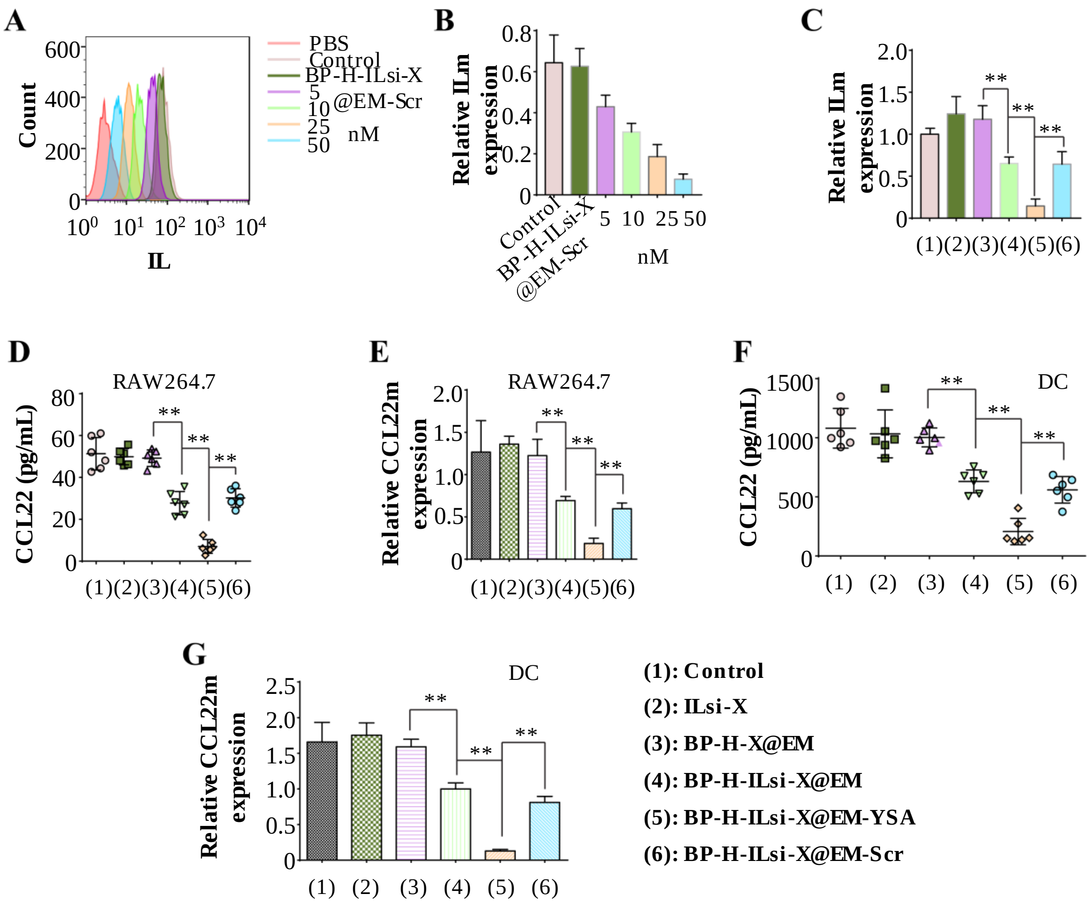

(A) *In vitro* measurement of IL secretion in MC-38 cells after treatment with BP-H-ILsi-X@EM-YSA nanosystem as a function of ILsi concentration in the nanosystem using flow cytometry. Relative levels of ILm in MC-38 cells determined by qRT-PCR after treatment with (B) the nanosystem as a function of ILsi concentration in the nanosystem or (C) the different configurations (1–6) (N = 6). CCL22 expressions and corresponding m levels in RAW264.7 macrophages (D, E) or BMDCs (F, G) after being co-cultured with MC-38 cells pretreated with the different configurations (1–6) (N = 6; $^{**}p < 0.01$).

Figure S8

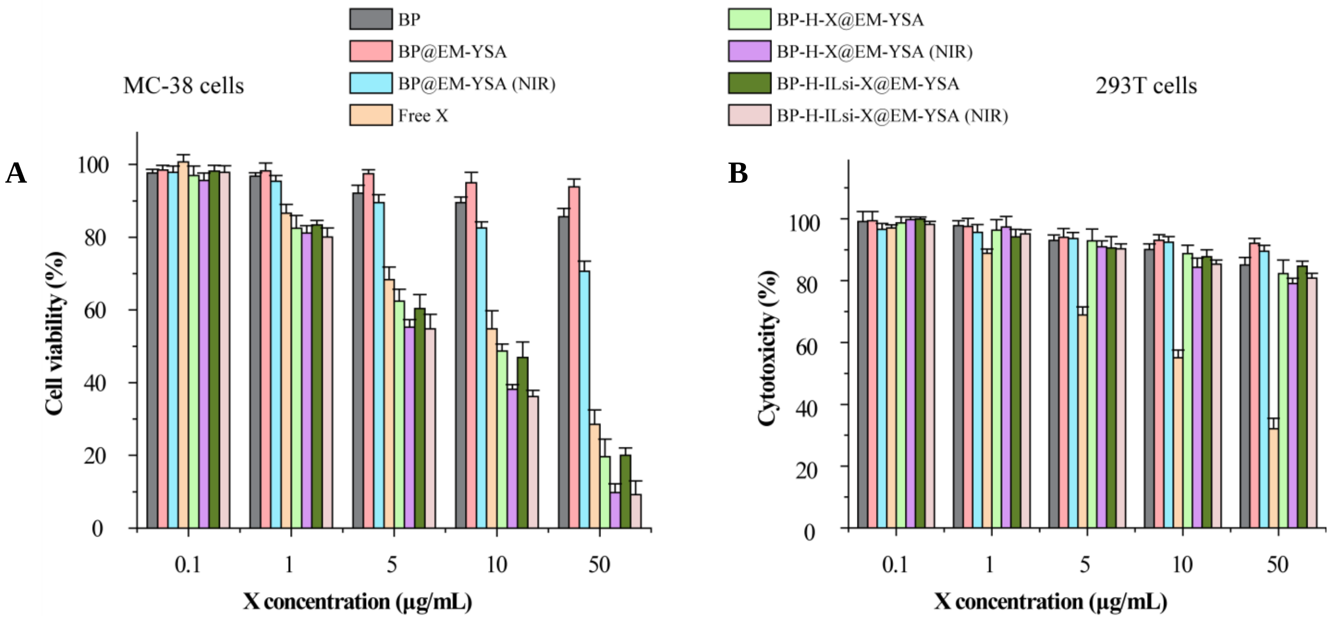

Viabilities of MC-38 (A) and 293T (B) cells incubated with BP, free X, BP@EM-YSA, BP-H-X@EM-YSA, and BP-H-ILsi-X@EM-YSA for 24 h in the absence and presence of NIR irradiation (808 nm, 0.5 W/cm^2, 5 min) using MTT assay (N = 6).

Figure S9

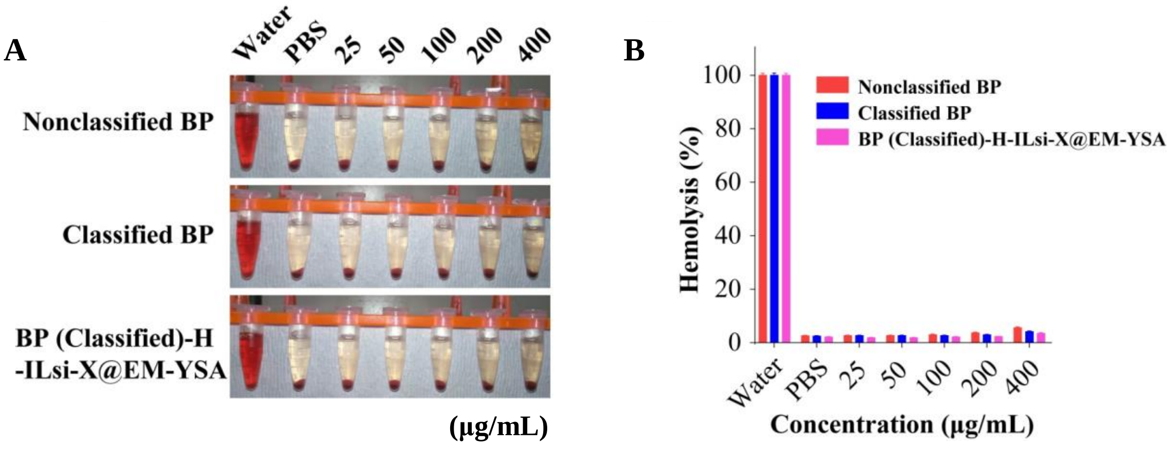

(A) Digital images and (B) percentages of EM hemolysis at different concentrations of nonclassified BP, classified BP, and BP (classified)-H-ILsi-X@EM-YSA (N = 3).

Figure S10

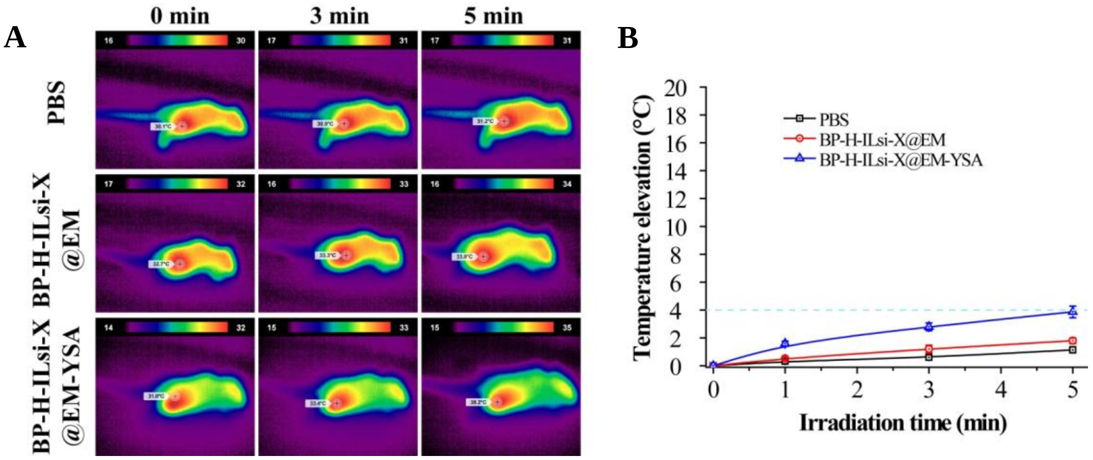

Photothermal contours of the treated (PBS, BP-H-ILsi-X@EM, BP-H-ILsi-X@EM-YSA) mice (A) and corresponding temperature elevation profiles in the tumor regions (B) during NIR irradiation (808 nm, 0.5 W/cm^2, 5 min, $N = 6$).

Figure S11

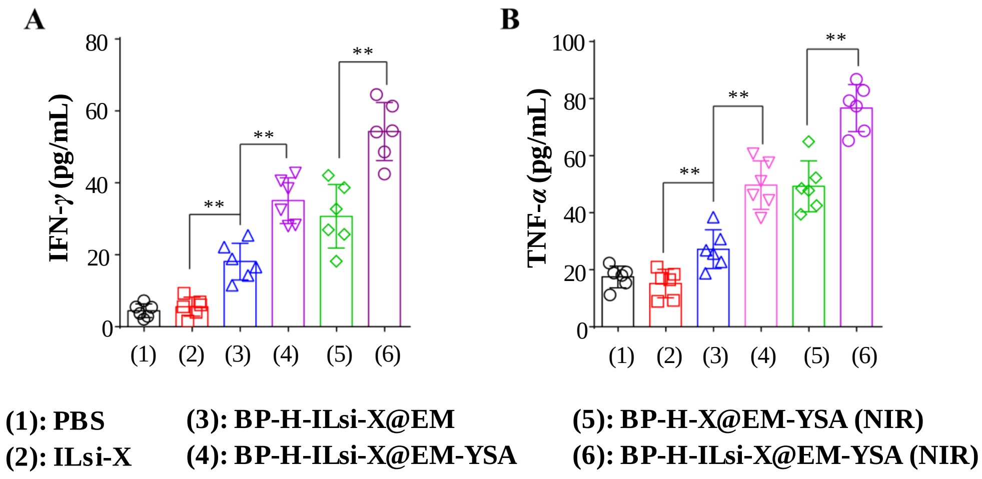

Mean serum levels of IFN-γ (A) and TNF-α (B) in mice treated with the different configurations (1–6) ($N = 6$; $^{**}p < 0.01$).

Figure S12

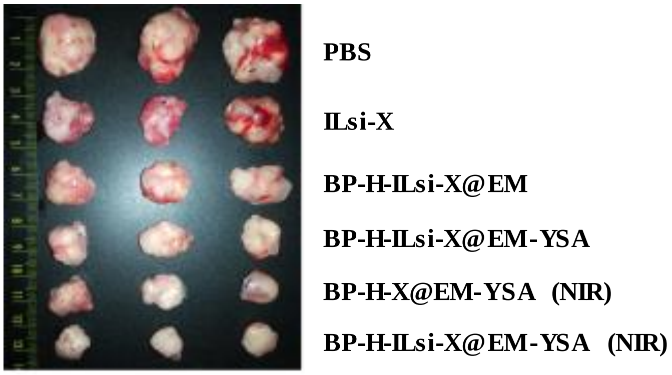

Representative digital images of tumor masses isolated from MC-38 tumor-bearing mice treated with the different configurations.

Figure S13

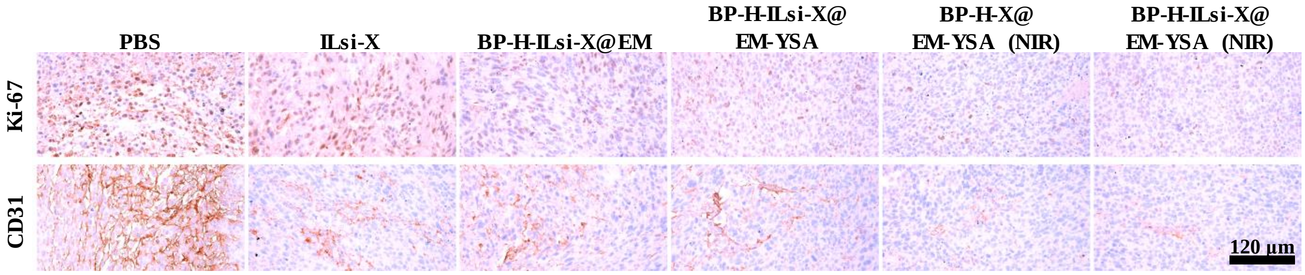

Immunohistochemical analysis of Ki-67 and CD31 in tumor tissues after treatment with the different configurations.

Figure S14

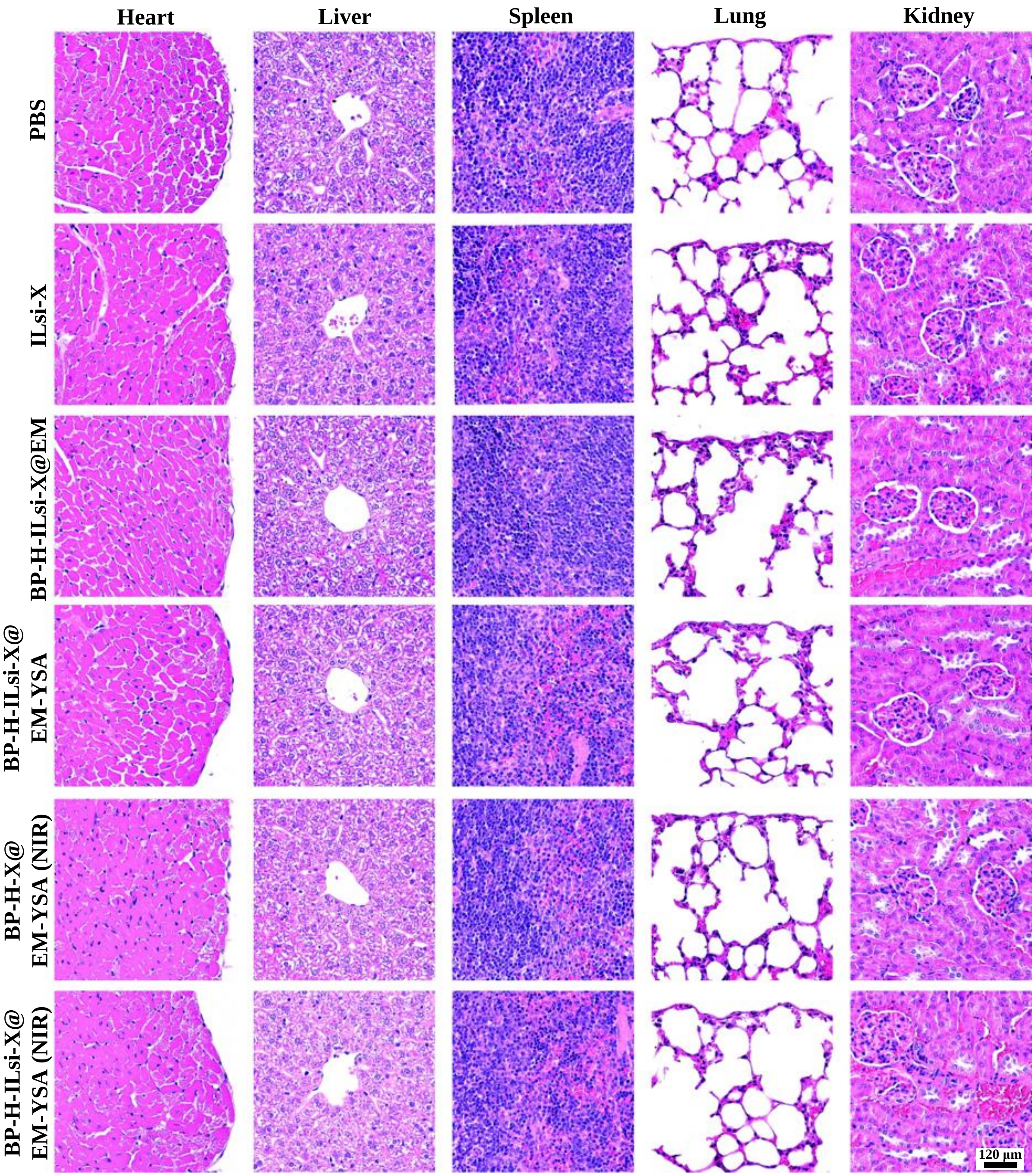

Representative H&E staining of the heart, liver, spleen, lung, and kidney in MC-38 tumor-bearing mice treated with the different configurations.

TABLE S1

Histomorphometrical analysis of tumor masses taken from MC-38 tumor-bearing C57BL/6 mice

Item / Group	Tumor cell volume (%/mm²)	Immunoreactive cell percentage (%/mm² of tumor mass)		Immunoreactive cell number (cells/mm² of tumor mass)	
		Ki-67	CD31 (PECAM-1)	$CD8^+$	Foxp3
**Control (G1)**	77.56±10.95	62.99±12.48	50.27±13.16	65.67±14.39	375.00±60.49
**Treatment**					
**G2**	76.95±11.34	58.06±13.54	47.96±11.65	69.33±13.19	363.67±48.90
**G3**	54.60±7.47[ab]	38.29±5.22[ab]	28.62±4.24[ab]	167.33±24.74[ab]	150.33±18.26[ab]
**G4**	39.37±6.69[abd]	29.96±2.47[abc]	20.40±2.32[abc]	237.33±35.55[abc]	88.50±15.90[abc]
**G5**	28.18±4.64[abce]	17.46±2.01[abce]	12.91±2.85[abce]	248.00±180.60[abce]	221.00±48.67[abce]
**G6**	15.38±3.85[abcef]	8.54±3.95[abcef]	5.13±1.62[abcef]	3052.33±944.75[abcef]	43.83±19.17[abcef]

Values are expressed as the mean ± SD of six tumor mass histological fields.

Treatment groups: G1 = PBS, G2 = ILsi-X, G3 = BP-H-ILsi@EM, G4 = BP-H-ILsi-X@EM-YSA, G5 = BP-H-X@EM-YSA (NIR), and G6 = BP-H-ILsi-X@EM-YSA (NIR).

PECAM-1: platelet/endothelial cell adhesion molecule-1.

[a] $p < 0.01$, as compared with G1 by Mann–Whitney (MW) test.
[b] $p < 0.01$, as compared with G2 by MW test.
[c] $p < 0.01$ and [d] $p < 0.05$, as compared with G3 by MW test.
[e] $p < 0.01$, as compared with G4 by MW test.
[f] $p < 0.01$, as compared with G5 by MW test.
